# Adenosine A2A Receptor Suppressed Astrocyte-Mediated Inflammation Through the Inhibition of STAT3/YKL-40 Axis in Mice With Chronic Cerebral Hypoperfusion-induced White Matter Lesions

**DOI:** 10.3389/fimmu.2022.841290

**Published:** 2022-02-14

**Authors:** Jichao Yuan, Lin Chen, Jie Wang, Simin Xia, Jialu Huang, Linke Zhou, Chengjian Feng, Xiaofei Hu, Zhenhua Zhou, Hong Ran

**Affiliations:** ^1^ Department of Neurology, Southwest Hospital, Third Military Medical University (Army Medical University), Chongqing, China; ^2^ Department of Medical Engineering, 958th Hospital of the People’s Liberation Army, Chongqing, China; ^3^ Department of Radiology, Southwest Hospital, Third Military Medical University (Army Medical University), Chongqing, China

**Keywords:** adenosine A2A receptor (ADORA2A), astrocyte, inflammation, YKL-40, white matter lesions

## Abstract

White matter lesions are an important pathological manifestation of cerebral small vessel disease, with inflammation playing a pivotal role in their development. The adenosine A2a receptor (ADORA2A) is known to inhibit the inflammation mediated by microglia, but its effect on astrocytes is unknown. Additionally, although the level of YKL-40 (expressed mainly in astrocytes) has been shown to be elevated in the model of white matter lesions induced by chronic cerebral hypoperfusion, the specific regulatory mechanism involved is not clear. In this study, we established *in vivo* and *in vitro* chronic cerebral hypoperfusion models to explore whether the ADORA2A regulated astrocyte-mediated inflammation through STAT3/YKL-40 axis and using immunohistochemical, western blotting, ELISA, PCR, and other techniques to verify the effect of astrocytes ADORA2A on the white matter injury. The *in vivo* experiments showed that activation of the ADORA2A decreased the expression of both STAT3 and YKL-40 in the astrocytes and alleviated the white matter injury, whereas its inhibition had the opposite effects. Similarly, ADORA2A inhibition significantly increased the expression of STAT3 and YKL-40 in astrocytes *in vitro*, with more proinflammatory cytokines being released by astrocytes. STAT3 inhibition enhanced the inhibitory effect of ADORA2A on YKL-40 synthesis, whereas its activation reversed the phenomenon. These results suggest that the activation of ADORA2A in astrocytes can inhibit the inflammation mediated by the STAT3/YKL-40 axis and thereby reduce white matter injury in cerebral small vessel disease.

## Introduction

Cerebral small vessel disease, an important cause of cognitive dysfunction, is an age-related condition that seriously affects the quality of life of afflicted patients (1). Because cerebral white matter lesions are the main pathological manifestation of this disease, finding ways to alleviate white matter injury would improve the related cognitive dysfunction (2). Inflammation is known to be the main causative factor of white matter injury (3). Not only microglia (4), astrocytes also play important roles in mediating the inflammation in the central nervous system (CNS) (5). However, role of astrocytes in the development of white matter lesions are not clear.

The adenosine A2a receptor (ADORA2A), a cell-surface receptor involved in the modulation of the inflammatory responses in cells, plays an important role in the pathological changes of the CNS. It has been reported that ADORA2A can regulate microglial remodeling in a mouse model of chronic anxiety, thereby reducing the anxiety-related behaviors in the mice (6). Our research group had previously demonstrated that bone marrow-derived ADORA2A could improve the cognitive functions of mice with chronic cerebral hypoperfusion (CCH)-induced white matter injury by inhibiting microglia-mediated inflammation (7). In recent years, many research groups have sought to clarify the role of the ADORA2A signaling pathway in astrocytes. For example, it was reported that activation of the ADORA2A signaling pathway in astrocytes could reduce neuronal apoptosis in ischemic stroke (8). Moreover, knockout of the *ADORA2A* gene in astrocytes caused disorders of glutamate metabolism, leading to cognitive dysfunction in patients with schizophrenia (9). Given that astrocytes are the main cells in deep white matter tissue, how they are regulated by ADORA2A in white matter lesions is worthy of further study.

Chitinase 3-like-1 (CHI3L1), also known as YKL-40, has been extensively studied as a cerebrospinal fluid biomarker for diseases such as Alzheimer’s disease and dementia (10). As a carbohydrate-binding lectin that mediates inflammation, YKL-40 is regulated by the circadian rhythm of astrocytes and increases the deposition of beta-amyloid during the inflammatory response, thereby accelerating the pathological process of Alzheimer’s disease (11). Notably, the inflammatory cytokine interleukin (IL)-1 can activate the signal transducer and activator of transcription 3 (STAT3) pathway to promote the secretion of YKL-40 from primary astrocytes cultured *in vitro* (12). Although YKL-40 is known to be expressed mainly in astrocytes in diseases involving white matter demyelination (13), the mechanism through which it is regulated in white matter lesions remains unknown.

Therefore, on the presumption that astrocyte-mediated inflammation is an important cause of white matter lesions and that the ADORA2A/STAT3/YKL-40 axis plays a pivotal role in this process, this study was carried out to verify these hypotheses. To this end, we established *in vitro* and *in vitro* models of white matter injury and applied an agonist and an inhibitor of ADORA2A and *ADORA2A*-knockout mice to determine the effects on white matter lesions. Then, an activator and an inhibitor of STAT3 were used to determine how these different interventions affect the expression of the downstream *YKL-40* gene and the astrocyte-mediated inflammation. Additionally, the mechanism through which ADORA2A alleviates CCH-induced white matter injury was investigated to identify possible targets that could be used for the clinical treatment of cerebral small vessel disease and thereby improve the quality of life of affected patients.

## Materials and Methods

### Animals and Drugs

The *ADORA2A*-knockout (gKO) mice used in the study were obtained from Dr. Jiang-Fan Chen (Molecular Neuropharmacology Laboratory, Boston University School of Medicine, Boston, MA, USA). Wild-type (WT) C57 mice were purchased from the Department of Laboratory Animal Science, Army Medical University, China. All mice in this study were 8–9 weeks old and weighed 25–28 g. All animal experiments and care were approved by the Third Military Medical University Committee on Ethics in the Care and Use of Laboratory Animals.

The ADORA2A-specific agonist CGS21680 (Cat. No. 1063) and -specific inhibitor SCH58261 (Cat. No. 2270) were purchased from Tocris Bioscience (Abingdon, UK). The STAT3-specific activator Colivelin (sc-361153) and -specific inhibitor Stattic (sc-202818) were purchased from Santa Cruz Biotechnology, Inc. (Dallas, TX, USA).

### Establishment of the Chronic Cerebral Hypoperfusion Model

A mouse model of chronic cerebral hypoperfusion (CCH) was established by referring to previously described methods (7). After placing the mouse under isoflurane inhalation anesthesia, the bilateral common carotid arteries were fully freed. Then, two 3-0 nylon sutures were loosely looped around the proximal and distal ends of the right common carotid artery, which was subsequently suspended using hemostatic forceps and placed in a special mini coil with an inner diameter of 1.8 mm. The coil was then carefully placed around the common carotid artery. Another coil was placed around the left common carotid artery in the same way 30 min later. The mice in the sham group were subjected to the same surgery except that the bilateral common carotid arteries were not surrounded with the coils. During the operation, the rectal temperature of the mouse was maintained at 36.5–37.5°C. Additionally, the 418-1 probe of a laser Doppler flow meter was fixed at the junction of the temporal squama and the greater wing of the sphenoid above the zygomatic arch to measure the cerebral blood flow and to evaluate the modeling throughout the entire surgical process.

### Experiment Grouping

The following five groups were created for the first part of the animal experiments: (1) sham group, (2) CCH group, (3) CCH+SCH58261 group, (4) CCH+CGS21680 group, and (5) CCH+CGS21680 (KO) group. For the first four groups, WT mice were modeled as described above and then randomly assigned to groups 1, 2, 3, and 4. The mice in the SCH58261 group were intraperitoneally injected with 0.1 mg/kg SCH58261, those in the CGS21680 group were intraperitoneally injected with 0.25 mg/kg CGS21680, and those in the CCH and sham groups were intraperitoneally injected with the same volume of dimethyl sulfoxide (DMSO) (14), respectively. For the fifth group, gKO mice were also modeled as described above and then intraperitoneally injected with 0.25 mg/kg CGS21680.

In the second part of the animal experiment, WT mice were modeled and divided into the following eight groups: (1) sham group, (2) CCH group, (3) CCH+Stattic group, (4) CCH+Colivelin group, (5) CCH+CGS21680 group, (6) CCH+CGS21680+Colivelin group, (7) CCH+ SCH58261 group, and (8) CCH+SCH58261+Stattic group. Stattic was intraperitoneally injected into the designated groups at a concentration of 2.5 mg/kg (15) and Colivelin likewise at a concentration of 1 mg/kg (16). All drugs were administered once every 24 h until the sudden death of the animals.

### Immumohistochemical Staining for Myelin Basic Protein

An the end of 2nd, 4th, and 6th weeks after CCH, five mice were taken from each group, deeply anesthetized with 1% sodium pentobarbital (60 mg/kg), and then perfused with 4% paraformaldehyde. Subsequently, the brain tissue was excised, fixed, dehydrated, and frozen. Then, the frozen brain sectioned (10-μm-thick slices) were incubated with 3% H_2_O_2_ for 10 min and blocked with goat serum for 30 min. Then, a primary antibody solution against myelin basic protein (MBP) (1:200 dilution; Abcam, Cambridge, MA, USA) was added for overnight incubation at 4°C, following which a sheep anti-rabbit IgG secondary antibody solution (1:200; Zhongshan Biotechnology, Beijing, China) was added for 30 min incubation at ambient temperature. Finally, nickel ammonium sulfate was used to enhance the color development, and Image Pro Plus software was used for semi-quantitative analysis of the staining results. A semi-quantitative analysis was used to assess the MBP^+^ cells as previously reported (14). Briefly, the MBP^+^ cells in a 0.5 mm × 0.5 mm width and 0.25 mm^2^/field of view were counted. The MBP^+^ cells counterstained with DAPI in nuclear were included for analysis.

### Primary Astrocyte Culture

Primary astrocytes were cultured according to previously described methods (17). The cerebral hemisphere of mice born within 3 days was excised and the pia mater was stripped off. The tissue was minced and then digested with 0.125% trypsin for 8 min, following which F12 complete medium containing 10% fetal bovine serum was added to stop the digestion. After centrifugation, the cell pellet was resuspended in F12 complete medium containing 100 μg/mL penicillin and 100 μg/mL streptomycin and transferred to a 75 mL culture flask for differential adhesion for 50 min. Then, the non-adherent cells were cultured for 12 h in an incubator at 37°C with 5% CO_2_. The medium was changed after the cells had adhered and again every 3 days. On the 9th day, the culture was shaken at 250 rpm for 18 h to remove the microglia and oligodendrocytes into the upper medium layer. Finally, astrocytes with a purity of more than 95% were obtained after 3 days of culture. After CGS21680 (100 nM), SCH58261 (50 nM) (18), Colivelin (10 nM) (19), and Stattic (50 nM) (20) had been added separately, the primary astrocytes were cultured for 72 h under mild oxygen-glucose deprivation (mOGD) conditions; that is, in low-glucose Dulbecco’s modified Eagle’s medium/F12 medium (1 g/L) in a hypoxia incubator (3% O_2_, 5% CO_2_, 92% N_2_). Subsequently the expression levels of the target factors were determined.

### Immunofluorescence

The animal tissue specimens and cell samples were fixed with 4% paraformaldehyde for 30 min, permeated with 0.1% Triton X-100 for 2 h, and blocked with 5% goat serum for 30 min (all carried out at ambient temperature). Then, the samples were incubated overnight at 4°C with primary antibodies against the following proteins: glial fibrillary acidic protein (GFAP) (1:1000 dilution; ab4674, abcam), STAT3 (1:100; Cat. No. 9145, Cell Signaling Technology (CST), Danvers, MA, USA), and YKL-40 (1:50; ab180569, abcam). Then, fluorescein-labeled secondary antibody and DAPI (4′,6-diamidino-2-phenylindole) stain solutions were added under dark conditions. Finally, the expression of the target proteins were detected under a confocal laser scanning microscope.

### Western Blot Analysis

Total proteins were extracted from the cells with RIPA lysis buffer, and the protein concentration was determined using a bicinchoninic acid kit. Then, the proteins were denatured by boiling the sample for 5 min. Thereafter, the proteins were separated by 10% sodium dodecyl sulfate-polyacrylamide gel electrophoresis at a constant voltage of 80 V for 100 min, with the sample loading volume calculated on the basis of a 20 μg loading amount. After electrophoresis, the protein bands were electrotransferred to a polyvinylidene difluoride membrane at a constant current of 200 mA for 100 min. Subsequently, the membrane was blocked for 2 h at ambient temperature with 5% bovine serum albumin and then incubated overnight at 4°C with primary antibodies against GFAP (1:10,000 dilution; abcam), STAT3 (1:1000; Cat. No. 12640, CST), phospho-STAT3 (1:1000; Cat. No. 9145, CST), and YKL-40 (1:500; abcam). This was followed by incubation with the secondary antibody for 1 h at ambient temperature. Finally, a gel imaging system was used to visualize the proteins.

### Polymerase Chain Reaction

Total RNA was extracted using the TRIzol reagent. Then, after the concentration and purity of the sample had been verified, it was subjected to polymerase chain reaction (PCR) quantitation using a TaKaRa quantitative PCR kit (TaKaRa, Bio, Shiga, Japan) according to the manufacturer’s instructions. The reverse transcription conditions were 42°C for 15 min and 85°C for 5 s, and the amplification conditions were 40 cycles of 95°C for 30 s, 95°C for 5 s, and 60°C for 30 s. The results were visualized using a gel imaging system. The forward and reverse primers for YKL-40 and STAT3 were 5ʹ-CCGTTCCTGCGTTCTTAT-3ʹ and 5ʹ-ACTGGTTGCCCTTGGTAG-3ʹ, 5ʹ-GGAGGAGTTGCAGCAAAAAG-3ʹ and 5ʹ-TGTGTTTGTGCCCAGAATGT5ʹ, respectively, and were designed and synthesized by Sangon Biotech (Shanghai) Co., Ltd. (Shanghai, China).

### Enzyme-Linked Immunosorbent Assay

After processing of the astrocytes, the enzyme-linked immunosorbent assay (ELISA) kit from eBioscience (San Diego, CA, USA) was used to detect the levels of inflammatory cytokines in the cell culture supernatant. Standards were prepared according to the kit instructions, and the supernatant samples were then added for incubation at 37°C for 30 min. After repeated washes with phosphate-buffered saline, the enzyme-labeled reagent was added for incubation at 37°C for 30 min. Finally, the stop solution was added to terminate the reaction, and the absorbance of the solution in each well was measured at 450 nm using a microplate reader.

### Statistical analysis

SPSS 22.0 was used for all data analyses and all data are presented as the mean ± standard deviation (SD). One-way analysis of variance was applied for comparing the data of multiple groups, and Tukey’s test was used for comparisons between groups. Differences with a *P* value of less than 0.05 were considered statistically significant.

## Results

### Activation of ADORA2A Reduced White Matter Injury in the Mice

**Figure 1:**
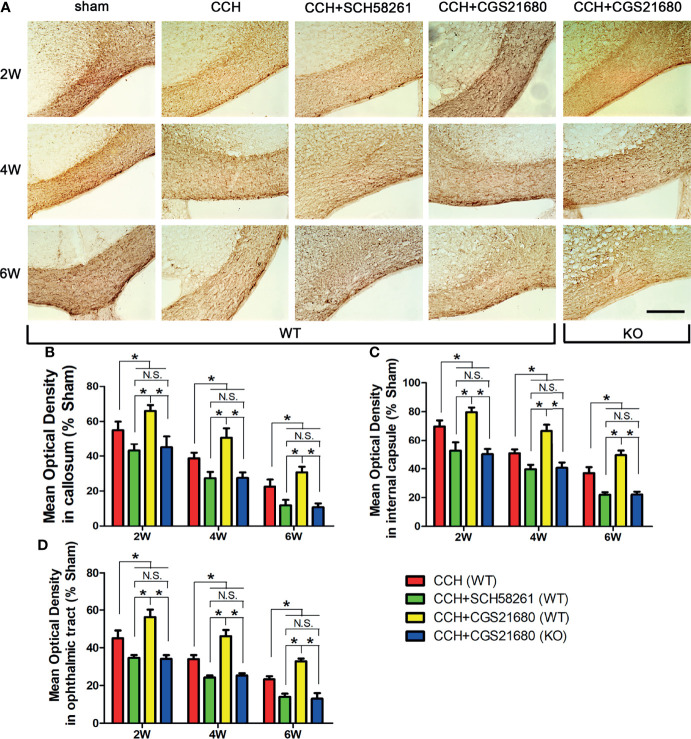
Activation of ADORA2A reduced white matter injury in the mouse model of CCH induced white matter lesions. **(A)** Detection of white matter injury in the corpus callosum using immumohistochemical staining for MBP after CCH. **(B–D)** Statistical analysis of the MBP absorbance in the corpus callosum **(B)**, internal capsule **(C)**, and optic tract **(D)** at the end of 2nd, 4th, and 6th week after CCH, respectively. These results suggest that hypoperfusion leads to white matter injury in a time-dependent manner, and the activation of ADORA2A could inhibit the injury caused by CCH. Scale bars = 50 μm; N = 6; N.S. indicated no significant difference, *P < 0.05.

To verify the effects of ADORA2A, we administered an agonist and an inhibitor of the receptor to mice with surgically induced CCH to evaluate the degree of white matter injury. The results of immunofluorescence (**Figure 1**) showed that the MBP absorbance was lower in the CCH group than that in the sham group and gradually decreased with prolongation of the ischemia time, confirming that CCH caused white matter injury in a time-dependent manner. Compared with that in the CCH group, the MBP absorbance was significantly lower in the SCH58261 group but significantly higher in the CGS21680 group, indicating that the activation of ADORA2A could inhibit the white matter injury induced by CCH. The MBP absorbance in the CGS21680 (KO) group was also lower than that in the CCH group, further confirming the protective role of ADORA2A against CCH-induced white matter injury.

### Activation of ADORA2A Inhibited YKL-40 Expression in Astrocytes

**Figure 2:**
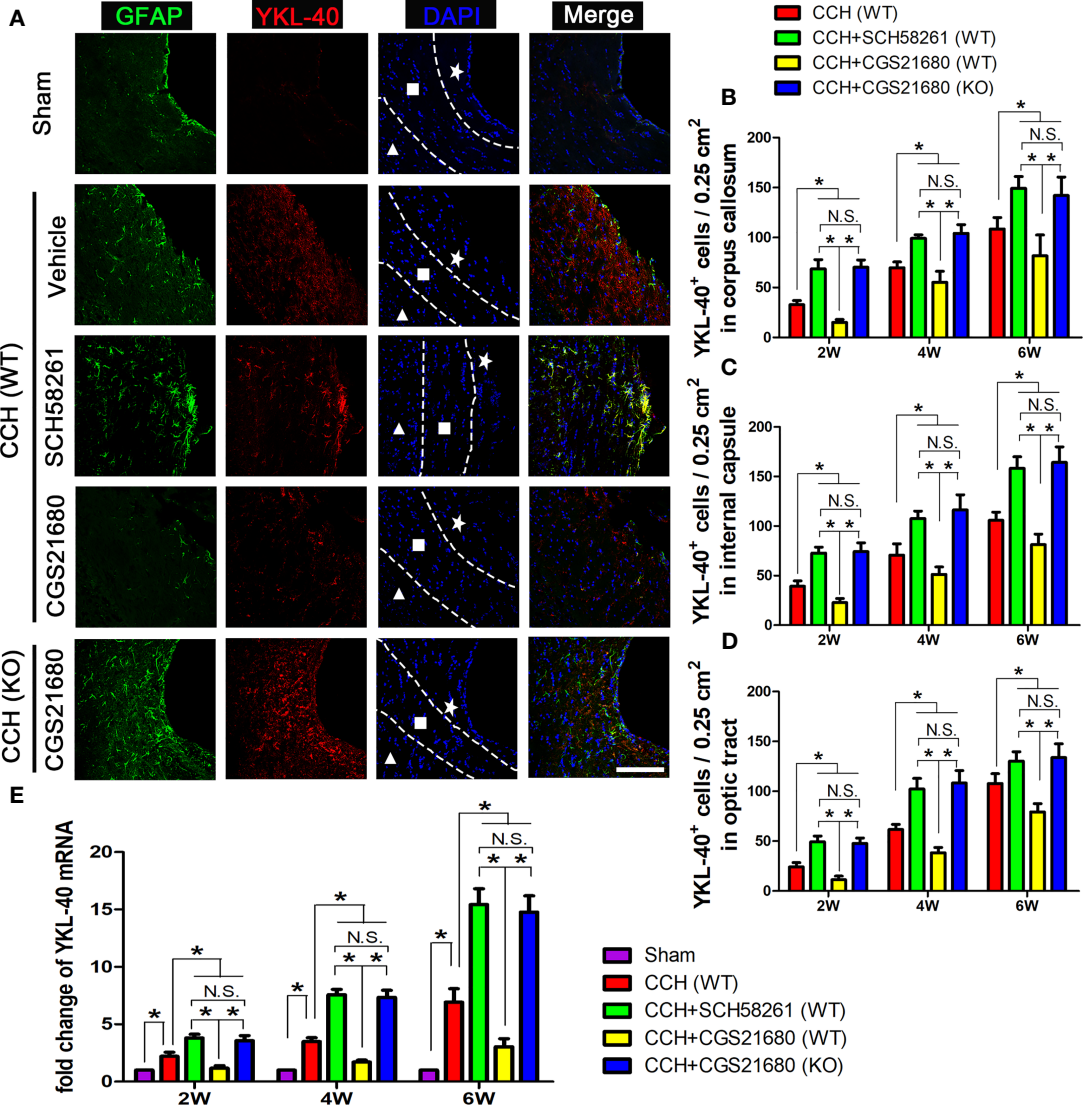
Activation of ADORA2A inhibited YKL-40 expression in astrocytes. **(A)** Immunofluorescence for astrocyte activation and YKL-40 expression at the end of 2nd week post CCH. The astrocyte-specific marker GFAP was labeled with Alexa Fluor 488 (green), while the YKL-40 was labeled with Alexa Fluor 555 (red). The white pentagram, the foursquare and the solid triangle indicates the corpus callosum area, the internal capsule area, and the optic tract area, respectively. **(B–D)** Statistical analysis of the number of YKL-40-positive cells in the corpus callosum **(B)**, internal capsule **(C)**, and optic tract **(D)** at the end of 2nd, 4th, and 6th week after CCH, respectively. **(E)** Levels of *YKL-40* mRNA in the cerebrum at the end of 2nd, 4th, and 6th week after CCH. The results verified that the activation of ADORA2A could inhibit the activation of astrocytes and reduce the expression of YKL-40 both in protein and mRNA levels. Scale bars = 20 μm; N = 6; N.S. indicated no significant difference, *P < 0.05.

To investigate how ADORA2A activation results in the reduction of white matter injury, we used immunofluorescence and PCR assays to detect the expression level of YKL-40 in the brain (**Figure 2A**). The immunofluorescence results showed that YKL-40 was barely expressed in the sham group, whereas its expression level was increased in the CCH group. Moreover, YKL-40 was mainly co-localized with GFAP, indicating that it was predominantly expressed in the astrocytes. Compared with the GFAP and YKL-40 levels in the CCH group, the expression in the SCH58261 group were significantly higher, whereas those in the CGS21680 group were significantly lower, indicating that the activation of ADORA2A could inhibit the activation of astrocytes and reduce the expression of YKL-40 (**Figures 2B–D**). The expression of GFAP and YKL-40 in the CGS21680 (KO) group were significantly higher than those in the CGS21680 (WT) group, further confirming that knockout of the *ADORA2A* gene promoted the CCH-induced activation of astrocytes and their expression of YKL-40. The PCR results showed the same expression trends for these target genes (**Figure 2E**). Taken together, these results verified that the activation of ADORA2A could inhibit astrocyte activation and decrease their expression of YKL-40.

### STAT3 Was Involved in the Adenosine ADORA2A Regulation of YKL-40 Expression

**Figure 3:**
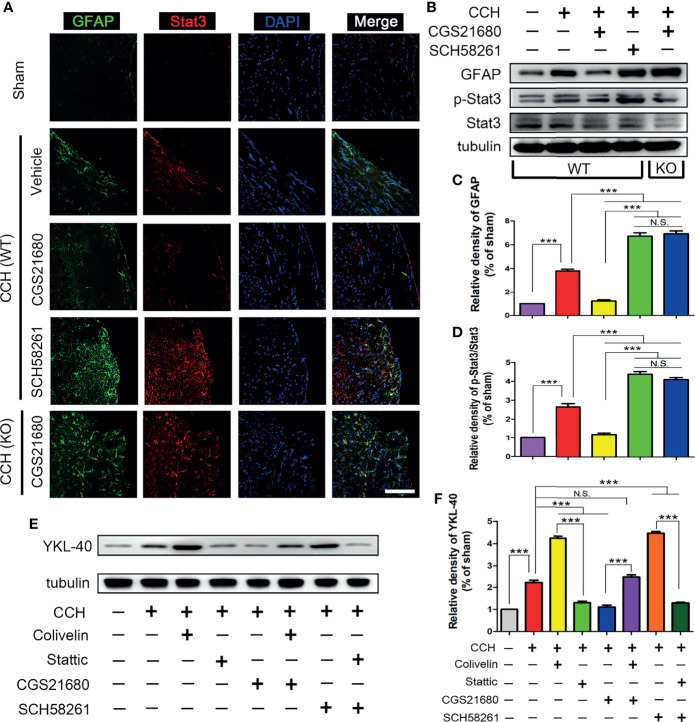
STAT3 was involved in the ADORA2A regulation of YKL-40 expression. **(A)** Immunofluorescence detection of astrocyte activation and STAT3 expression in the corpus callosum after CCH. **(B)** Western blot of the GFAP and STAT3 protein levels with the ADORA2A intervention. **(C, D)** Statistical analysis of the western blot results for GFAP and STAT3. **(E, F)** Western blot of the downstream YKL-40 protein level with ADORA2A and STAT3 intervention **(E)**, and statistical analysis **(F)** of the western blot results. Scale bars = 20 μm; N = 5; N.S. indicated no significant difference, ***P < 0.001.

To further evaluate the mechanism through which ADORA2A regulated YKL-40 expression, we evaluated the expression of STAT3 after CCH in mice. The immunofluorescence results showed the expression of STAT3 was higher in the CCH group than that in the sham group, suggesting that STAT3 was involved in the CCH-induced white matter lesions (**Figure 3A**). Compared with the GFAP and STAT3 levels in the CCH group, the expression in the SCH58261 group were significantly higher and those in the CGS21680 group were significantly lower, indicating that ADORA2A prevented the expression of STAT3 mediated by activation of the astrocytes (**Figures 3B–D**). The expression of STAT3 in the CGS21680 (KO) group was significantly higher than that in the CGS21680 (WT) group, further confirming that ADORA2A inhibited the expression of STAT3 in astrocytes.

Interestingly, the expression trend of STAT3 was consistent with that of YKL-40, suggesting that there was a certain causal relationship between them. Therefore, we used a STAT3 inhibitor (Stattic) and a STAT3 activator (Colivelin) on the WT mice to further evaluate these regulatory effects on YKL-40 expression. The western blot results revealed that the expression of YKL-40 was increased in the Colivelin group and decreased in the Stattic group relative to that in the CCH group, confirming that STAT3 could promote the expression of YKL-40 (**Figures 3E, F**). The expression of YKL-40 was significantly different between the CGS21680 and CGS21680+Colivelin groups, as were the levels between the SCH58261 and the SCH58261+Stattic groups, indicating that the activation of STAT3 could reverse the inhibitory effect of ADORA2A on YKL-40 expression. These results clearly demonstrate that ADORA2A reduces the synthesis of YKL-40 by inhibiting the expression of STAT3.

### ADORA2A Regulated the STAT3/YKL-40 Signaling Pathway in Astrocytes

**Figure 4:**
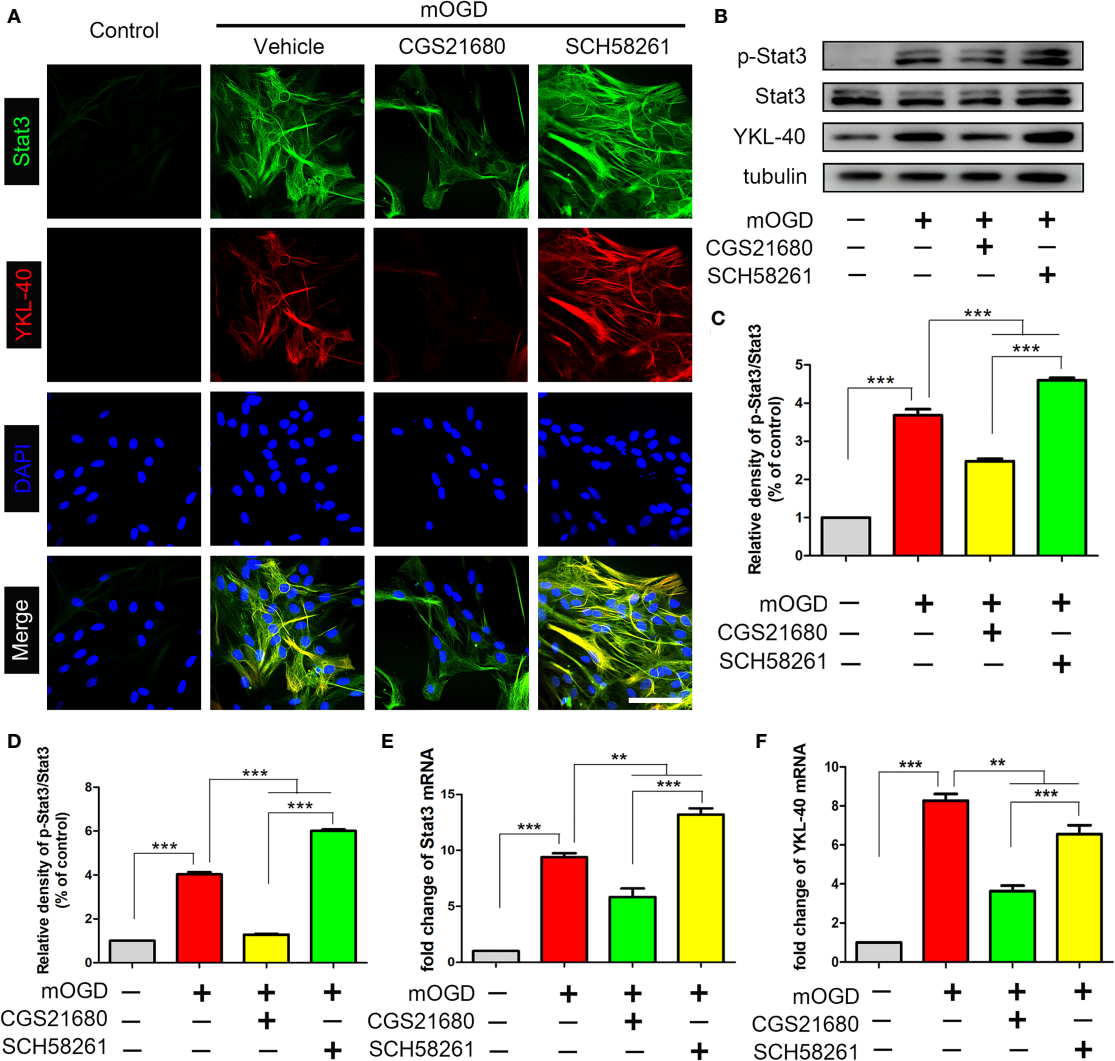
Activation of ADORA2A inhibited the expression of STAT3 and YKL-40 in astrocytes *in vitro*. **(A)** Immunofluorescence for STAT3 and YKL-40 expression with the regulation of ADORA2A in primary astrocytes with mOGD. **(B)** Western blot of the STAT3 and YKL-40 protein levels with the ADORA2A intervention. **(C, D)** Statistical analysis of the western blot results for STAT3 and YKL-40. **(E, F)** Real-time PCR assay of the *STAT3* and *YKL-40* mRNA levels with the ADORA2A intervention. Scale bars = 50 μm; N = 5; **P < 0.01, ***P < 0.001.

We used primary astrocytes *in vitro* to further verify the effect of ADORA2A on the STAT3/YKL-40 axis. The immunofluorescence results showed that after the mOGD treatment, astrocytes were activated and the expression of STAT3 and YKL-40 were increased, confirming that the two proteins were involved in the activation of astrocytes caused by chronic ischemia (**Figure 4A**). However, after activating ADORA2A with CGS21680 in the astrocytes, the expression of STAT3 and YKL-40 were decreased. By contrast, the inhibition of ADORA2A with SCH58261 in astrocytes led to increased expression of STAT3 and YKL-40. The same results were observed in the western blot (**Figures 4B–D**) and PCR (**Figures 4E, F**) experiments. These findings verified that ADORA2A regulated the STAT3/YKL-40 axis in astrocytes.

**Figure 5:**
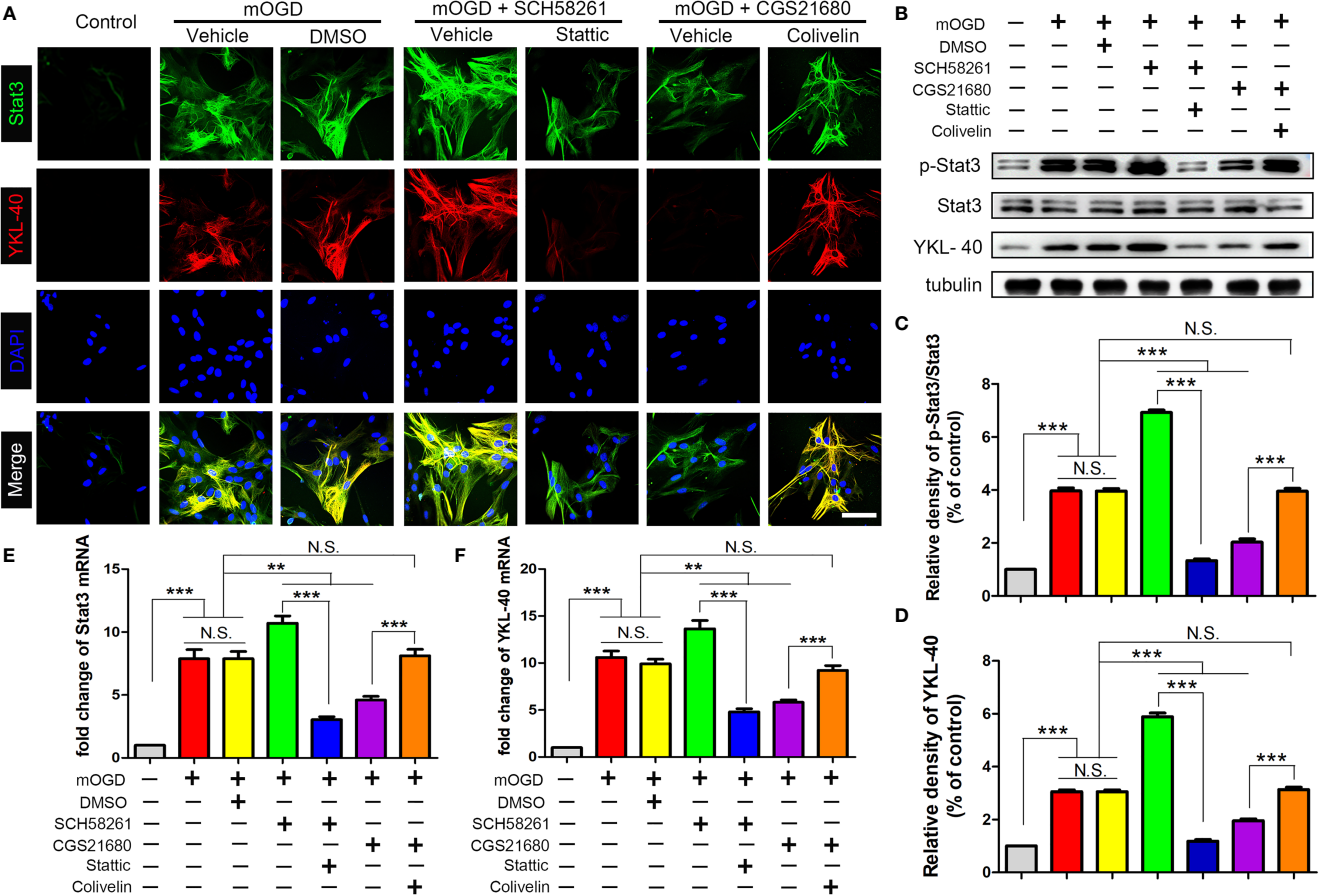
ADORA2A inhibited YKL-40 expression through the STAT3 signaling pathway. **(A)** Immunofluorescence detection of STAT3 and YKL-40 expression under different interventions in primary astrocyte with mOGD. **(B)** Western blot of the STAT3 and YKL-40 protein levels in astrocytes with the ADORA2A and STAT3 intervention. **(C, D)** Statistical analysis of the western blot results for STAT3 and YKL-40. **(E, F)** Real-time PCR quantitation of the *STAT3* and *YKL-40* mRNA levels in astrocytes under the regulation of ADORA2A and STAT3. Scale bars = 50 μm; N = 5; N.S. indicated no significant difference, **P < 0.01 ***P < 0.001.

After inhibited ADORA2A with SCH58261, the astrocyte bodies became swollen and their axons were elongated and interwoven into a network, suggesting that the cells had been further activated (**Figure 5**). Additionally, the expression of STAT3 and YKL-40 were increased. By contrast, astrocyte activation was significantly suppressed with CGS21680 intervention, further confirming that ADORA2A inhibited astrocyte activation. Moreover, compared with that in the SCH58261 group, the expression of YKL-40 was decreased by the further inhibition of STAT3 (SCH58261+ Stattic group). By contrast, the further activation of STAT3 (CGS21680 + Colivelin group) reversed the inhibitory effect of adenosine ADORA2A on YKL-40 synthesis that was observed in the CGS21680 group. These results verified that ADORA2A could downregulate YKL-40 synthesis by suppressing STAT3 expression, thereby inhibiting the activation of astrocytes.

### ADORA2A Inhibited Astrocyte-Mediated Inflammation Through STAT3/YKL-40 Axis

**Figure 6:**
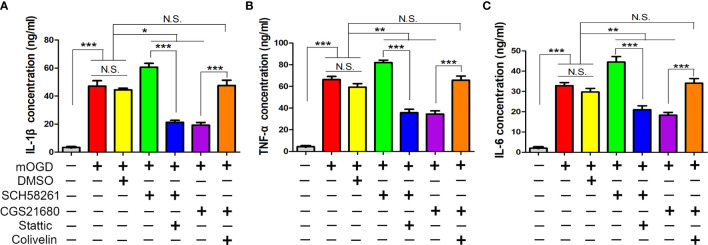
ADORA2A inhibited astrocyte-mediated inflammation through STAT3/YKL-40 axis. ELISA was used to detect the expression of the inflammatory cytokines IL-1β **(A)**, TNF-α **(B)**, and IL-6 **(C)** in the supernatant of primary astrocytes cultured with different treatments. The results confirmed that ADORA2A inhibited astrocyte-mediated inflammation through the STAT3/YKL-40 signaling pathway. N = 5; N.S. indicated no significant difference, *P < 0.05, **P < 0.01 ***P < 0.001.

Furthermore, we used ELISA to measure the astrocyte-mediated inflammatory response regulated by ADORA2A. The mOGD treatment promoted the IL-1β, tumor necrosis factor-alpha (TNF-α), and IL-6 secretion in the supernatant of the astrocyte, indicating that the low-oxygen conditions had activated the astrocytes and induced them to release inflammatory cytokines for mediating inflammation (**Figure 6**). The expression of these cytokines in the SCH58261 and CGS21680 groups were significantly different from those in the CCH group and indicated that ADORA2A could inhibit astrocyte-mediated inflammation. The further inhibition of STAT3 (SCH58261 + Stattic group) reduced the cytokine secretion when compared with that in the SCH58261 group. By contrast, the further activation of STAT3 (CGS21680 + Colivelin group) reversed the inhibitory effect of ADORA2A on inflammation that was seen in the CGS21680 group. These results provided further evidence that ADORA2A inhibits astrocyte-mediated inflammation through STAT3/YKL-40 axis.

## Discussion

Until now, the pathogenesis of white matter lesions has remained unclear, and the role of astrocytes in this process has been even less studied. We established a model of CCH-induced white matter lesions to confirm our hypothesis that the activation of ADORA2A could inhibit the expression of STAT3 and YKL-40 in astrocytes and thereby alleviated white matter injury. Primary astrocytes were also used to establish a model of chronic hypoxia to further verify that ADORA2A activation could inhibit the activation of astrocytes and downregulate their mediation of inflammation. The STAT3 inhibitor could enhance the effects of ADORA2A in inhibiting the astrocytes and reducing YKL-40 expression, whereas the STAT3 activator could reverse this phenomenon. Our results confirmed that ADORA2A inhibited the astrocyte-mediated inflammation through the STAT3/YKL-40 axis, thereby alleviated the white matter lesions induced by CCH.

ADORA2A is widely expressed in the CNS. In addition to regulating glutamate metabolism, it can also regulate immune responses. It was reported that the ADORA2A signaling pathway and the dopamine receptor signaling pathway could competitively regulate microglia-mediated inflammation and improve cognitive dysfunction in patients with Parkinson’s disease by regulating the immune microenvironment (21). Our research team also confirmed that the activation of ADORA2A in bone marrow-derived cells could reduce white matter injury in the corpus callosum and improve the cognitive dysfunction caused by CCH (7, 22). Because astrocytes are the most abundant cells in the CNS and are known to mediate inflammation in many diseases (23, 24), they have received extensive attention in the clinical imaging diagnosis of cerebral small vessel disease (25). However, their pathogenic mechanism in the development of white matter lesions is not clear. It was reported that upregulating the expression of ADORA2A in astrocytes could promote the activation and proliferation, and the release of inflammatory factors (26). It was also reported that ADORA2A could inhibit astrocyte-mediated inflammation through anti-inflammatory and antioxidative activities (27). These studies suggest that ADORA2A in astrocytes may play different biological functions in different disease models, and its role in cerebral small vessel disease is worthy of in-depth study.

To confirm the role of astrocyte ADORA2A in cerebral small vessel disease, we established a model of CCH-induced white matter injury and found that chronic hypoxia caused an increase in GFAP expression in the astrocytes and a significant decrease in the MBP absorbance in deep white matter tissue, such as the corpus callosum, internal capsule, and optic tract. At the end of 6th week, the MBP absorbance was further decreased compared with that at the end of 2nd week, indicating that chronic hypoxia leads to white matter lesions in a time-dependent manner. After treatment with the ADORA2A agonist CGS21680, the expression of GFAP in the astrocytes was significantly suppressed, whereas that of MBP absorbance was significantly increased. By contrast, treatment of ADORA2A inhibitor SCH58261 led to the increased GFAP expression and decreased MBP absorbance, further confirming that ADORA2A could inhibit the activation of astrocytes and reduce the white matter lesions induced by CCH. The increased secretion of the proinflammatory cytokines IL-1β, TNF-α, and IL-6 in astrocytes under mOGD also confirmed that chronic hypoxia could increase synthesis of these inflammatory factors by the astrocytes. CGS21680 reduced the expression of the three cytokines in the supernatant of astrocyte, whereas SCH58261 had the opposite effect. These results provide further evidence that ADORA2A inhibits astrocyte-mediated inflammation in CCH-induced white matter lesions.

YKL-40, which is a biomarker of inflammation, has been widely used in clinical practice as a relatively reliable diagnostic marker in various inflammatory diseases, such as non-alcoholic steatohepatitis and liver fibrosis (28), asthma and chronic obstructive pulmonary disease (29), and chronic kidney disease (30). In neurological diseases, such as Alzheimer’s disease (31), Huntington’s disease (32), and other neurodegenerative dementias (33), the level of YKL-40 in cerebrospinal fluid is elevated, which highly suggests that this protein is closely related to cognitive dysfunction. Cerebral small vessel disease is the most important cause of cognitive dysfunction, but the role of YKL-40 in the disease is not clear. Our study confirmed that the expression of YKL-40 in astrocytes was increased in CCH-induced white matter lesions, and inhibited by ADORA2A, activation, suggesting that YKL-40 mediates the white matter injury after CCH. To further explore how ADORA2A regulates YKL-40, we investigated the expression of STAT3 in astrocyte and found that the trends were consistent with those of YKL-40, where the activation of ADORA2A could inhibit the expression of STAT3. The STAT3 inhibitor Stattic enhanced the inhibitory effect of ADORA2A on YKL-40, and treatment with the STAT3 activator Colivelin abolished the phenomenon. These data confirmed that ADORA2A downregulated the expression of YKL-40 in astrocytes by inhibiting the STAT3 signaling pathway. STAT3, a nuclear transcription factor, is widely distributed in different types of cells and tissues. After its phosphorylation and dimerization, it enters the nucleus to regulate the expression of target genes and participates in the regulation of various physiological functions, such as cell growth, differentiation, and apoptosis. It is also closely related to inflammation, tumors, and the immune response. It has been shown that the activation of STAT3 upregulates the expression of YKL-40, thereby promoting the occurrence and progression of lung cancer (34, 35). Our study demonstrated that promoting the phosphorylation of STAT3 enhanced the activation of astrocytes, leading to chronic inflammatory conditions. Therefore, the ADORA2A/STAT3/YKL-40 axis in astrocytes plays an important role in chronic white matter injury.

## Conclusions

The inflammatory response mediated by astrocytes is an important cause of white matter lesions in cerebral small vessel disease. In this study, we found that chronic hypoxia promoted the activation of astrocytes by increased STAT3 and YKL-40 expression. Increased ADORA2A could inhibit astrocyte activation in the model of CCH-induced white matter lesions and suppress the inflammatory response, thereby alleviating the white matter injury caused by chronic hypoxia. The mechanism for ADORA2A protected against white matter injury was mainly through inhibition of the STAT3/YKL-40 axis. Our study provides a new potential target, ADORA2A, for the treatment of cerebral small vessel disease in clinical practice.

## Data Availability Statement

The original contributions presented in the study are included in the article/**Supplementary Material**. Further inquiries can be directed to the corresponding authors.

## Ethics Statement

The animal study was reviewed and approved by Third Military Medical University Committee on Ethics in the Care and Use of Laboratory Animals. Written informed consent was obtained from the owners for the participation of their animals in this study.

## Author Contributions

This study was based on the original idea of HR and ZZ. JY, LC, JW, SX, JH, and LZ conducted the experiments. XH and CF performed data analyses. JY and XH drafted the manuscript. All authors read and approved the final manuscript.

## Funding

This work was supported by grants from Chongqing Natural Science Foundation (cstc2018jcyjAX0203), National Natural Science Foundation of China (No. 81701146), and the Program for Distinguished Young Scholars of TMMU.

## Conflict of Interest

The authors declare that the research was conducted in the absence of any commercial or financial relationships that could be construed as a potential conflict of interest.

## Publisher’s Note

All claims expressed in this article are solely those of the authors and do not necessarily represent those of their affiliated organizations, or those of the publisher, the editors and the reviewers. Any product that may be evaluated in this article, or claim that may be made by its manufacturer, is not guaranteed or endorsed by the publisher.
