# Corrigendum: Adenosine A2A Receptor Suppressed Astrocyte-Mediated Inflammation through the Inhibition of STAT3/YKL-40 Axis in Mice with Chronic Cerebral Hypoperfusion-Induced White Matter Lesions

**DOI:** 10.3389/fimmu.2022.883172

**Published:** 2022-03-22

**Authors:** Jichao Yuan, Lin Chen, Jie Wang, Simin Xia, Jialu Huang, Linke Zhou, Chengjian Feng, Xiaofei Hu, Zhenhua Zhou, Hong Ran

**Affiliations:** ^1^Department of Neurology, Southwest Hospital, Third Military Medical University (Army Medical University), Chongqing, China; ^2^Department of Medical Engineering, 958th Hospital of the People’s Liberation Army, Chongqing, China; ^3^Department of Radiology, Southwest Hospital, Third Military Medical University (Army Medical University), Chongqing, China

**Keywords:** adenosine A2A receptor (ADORA2A), astrocyte, inflammation, YKL-40, white matter lesions

In the original article, it has been brought to the authors’ attention that there was a mistake in **Figure 1** as published. One image (sham group, 6w) was mistakenly chosen from the group named with sham-4w during the figure preparation. The corrected **Figure 1** appears below.

**Figure 1:**
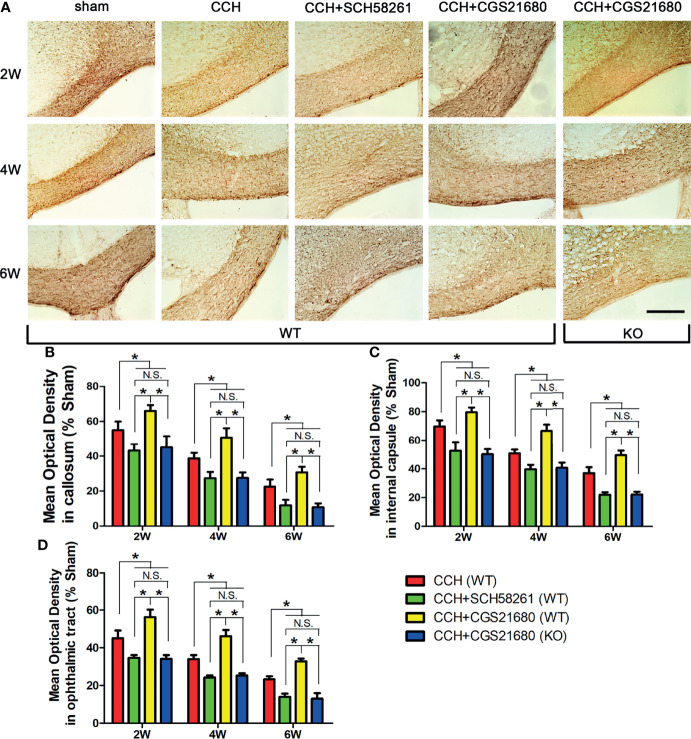
Activation of ADORA2A reduced white matter injury in the mouse model of CCH induced white matter lesions. **(A)** Detection of white matter injury in the corpus callosum using immumohistochemical staining for MBP after CCH. **(B–D)** Statistical analysis of the MBP absorbance in the corpus callosum **(B)**, internal capsule **(C)**, and optic tract **(D)** at the end of 2nd, 4th, and 6th week after CCH, respectively. These results suggest that hypoperfusion leads to white matter injury in a time-dependent manner, and the activation of ADORA2A could inhibit the injury caused by CCH. Scale bars = 50 μm; N = 6; N.S. indicated no significant difference, *P < 0.05.

In the original article, it has been brought to the authors’ attention that there was a mistake in **Figure 2** as published. One image (CCH (WT) + Vehicle) was mistakenly duplicated from another image (CCH (KO) + CGS21680) during the figure preparation. The corrected **Figure 2** appears below.

**Figure 2:**
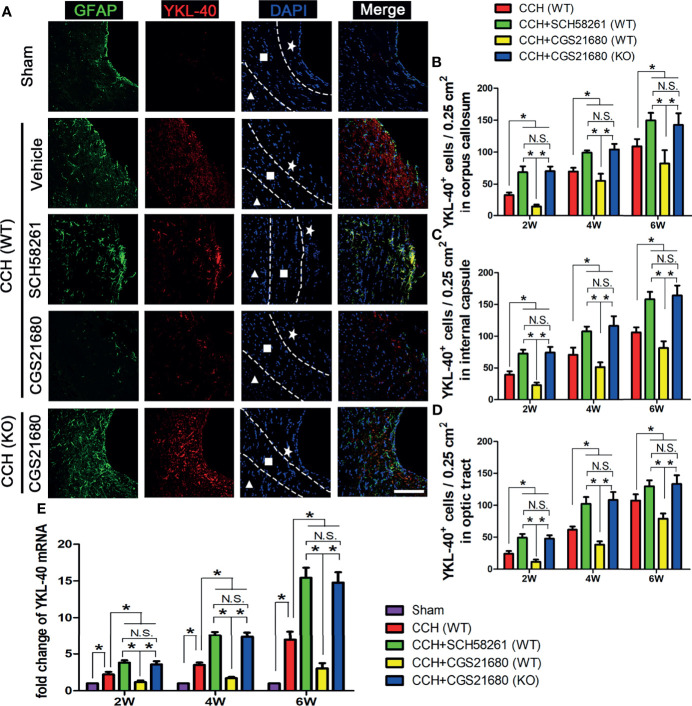
Activation of ADORA2A inhibited YKL-40 expression in astrocytes. **(A)** Immunofluorescence for astrocyte activation and YKL-40 expression at the end of 2nd week post CCH. The astrocyte-specific marker GFAP was labeled with Alexa Fluor 488 (green), while the YKL-40 was labeled with Alexa Fluor 555 (red). The white pentagram, the foursquare and the solid triangle indicates the corpus callosum area, the internal capsule area, and the optic tract area, respectively. **(B–D)** Statistical analysis of the number of YKL-40-positive cells in the corpus callosum **(B)**, internal capsule **(C)**, and optic tract **(D)** at the end of 2nd, 4th, and 6th week after CCH, respectively. **(E)** Levels of YKL-40 mRNA in the cerebrum at the end of 2nd, 4th, and 6th week after CCH. The results verified that the activation of ADORA2A could inhibit the activation of astrocytes and reduce the expression of YKL-40 both in protein and mRNA levels. Scale bars = 20 μm; N = 6; N.S. indicated no significant difference, *P < 0.05.

In the original article, it has been brought to the authors’ attention that there was a mistake in **Figure 3** as published. One image (CCH (WT) + CGS21680) was mistakenly duplicated from another image (CCH (WT) + SCH58261) during the figure preparation. The corrected **Figure 3** appears below.

**Figure 3:**
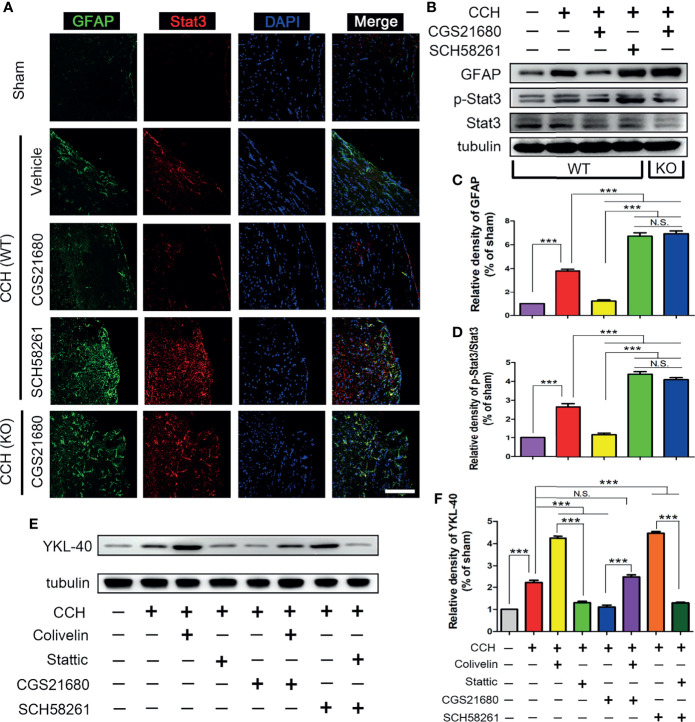
STAT3 was involved in the ADORA2A regulation of YKL-40 expression. **(A)** Immunofluorescence detection of astrocyte activation and STAT3 expression in the corpus callosum after CCH. **(B)** Western blot of the GFAP and STAT3 protein levels with the ADORA2A intervention. **(C, D)** Statistical analysis of the western blot results for GFAP and STAT3. **(E, F)** Western blot of the downstream YKL-40 protein level with ADORA2A and STAT3 intervention **(E)**, and statistical analysis **(F)** of the western blot results. Scale bars = 20 μm; N = 5; N.S. indicated no significant difference, ***P < 0.001.

The authors apologize for these errors and state that this does not change the scientific conclusions of the article in any way. The original article has been updated.

## Publisher’s Note

All claims expressed in this article are solely those of the authors and do not necessarily represent those of their affiliated organizations, or those of the publisher, the editors and the reviewers. Any product that may be evaluated in this article, or claim that may be made by its manufacturer, is not guaranteed or endorsed by the publisher.

