# Supplementary material for: Adenosine A2A Receptor Suppressed Astrocyte-Mediated Inflammation Through the Inhibition of STAT3/YKL-40 Axis in Mice With Chronic Cerebral Hypoperfusion-induced White Matter Lesions
Source: Front Immunol. 2022 Feb 14;13:841290. doi: 10.3389/fimmu.2022.841290 (PMC8882648; doi:10.3389/fimmu.2022.841290)
Supplement: Supplementary file 1 [file DataSheet_1.pdf]

Supplemental figure 1: Original data for the statistical analysis of the MBP absorbance in the corpus callosum at the end of 2nd week after CCH (for figure 1B).

2w original data	CCH	CCH+ SCH58261	CCH+ CGS21680	CCH+ CGS21680 (KO)
corpus callosum	54	43	66	46
	59	42	64	43
	62	48	62	38
	55	41	71	52
	48	47	64	53
	52	38	69	38

	CCH	CCH+ SCH58261	CCH+ CGS21680	CCH+ CGS21680 (KO)		
Mean ± SD	55.00 ± 4.98	43.17 ± 3.76	66.00 ± 3.41	45.00 ± 6.57		
Normality: Shapiro-Wilk normality test (W) P	(0.99) 0.98	(0.95) 0.74	(0.94) 0.63	(0.89) 0.30		
Homogeneity of Variances: Levene Statistic					F(3,20)=1.404	p=0.271
One way ANOVA					F=28.299	p=0.000

Multiple Comparisons	CCH vs. CCH+ SCH58261	CCH vs. CCH+ CGS21680	CCH vs. CCH+ CGS21680(KO)
Tukey HSD	p=0.002	p=0.004	p=0.009
LSD	p=0.000	p=0.001	p=0.002
	CCH+ SCH58261 vs. CCH+ CGS21680	CCH+ SCH58261 vs. CCH+ CGS21680 (KO)	CCH+ CGS21680 vs. CCH+ CGS21680 (KO)
Tukey HSD	p=0.000	p=0.912	p=0.000
LSD	p=0.000	p=0.519	p=0.000

Supplemental figure 2: Original data for the statistical analysis of the MBP absorbance in the corpus callosum at the end of 4th week after CCH (for figure 1B).

4w original data	CCH	CCH+ SCH58261	CCH+ CGS21680	CCH+ CGS21680 (KO)
corpus callosum	42	25	47	32
	37	32	57	24
	39	24	47	24
	43	28	45	27
	35	24	57	29
	36	31	52	29

	CCH	CCH+ SCH58261	CCH+ CGS21680	CCH+ CGS21680 (KO)		
Mean ± SD	38.67 ± 3.27	27.33 ± 3.56	50.83 ± 5.31	27.50 ± 3.15		
Normality: Shapiro-Wilk normality test (W) P	(0.92) 0.51	(0.86) 0.18	(0.85) 0.17	(0.91) 0.46		
Homogeneity of Variances: Levene Statistic					F(3,20)=1.948	p=0.154
One way ANOVA					F=48.791	p=0.000

Multiple Comparisons	CCH vs. CCH+ SCH58261	CCH vs. CCH+ CGS21680	CCH vs. CCH+ CGS21680(KO)
Tukey HSD	p=0.000	p=0.000	p=0.000
LSD	p=0.000	p=0.000	p=0.000

	CCH+ SCH58261 vs. CCH+ CGS21680	CCH+ SCH58261 vs. CCH+ CGS21680 (KO)	CCH+ CGS21680 vs. CCH+ CGS21680 (KO)
Tukey HSD	p=0.000	p=1.000	p=0.000
LSD	p=0.000	p=0.942	p=0.000

Supplemental figure 3: Original data for the statistical analysis of the MBP absorbance in the corpus callosum at the end of 6th week after CCH (for figure 1B).

6w original data	CCH	CCH+ SCH58261	CCH+ CGS21680	CCH+ CGS21680 (KO)
corpus callosum	21	16	33	9
	26	10	30	13
	24	12	32	12
	21	14	35	7
	27	12	27	12
	16	7	27	11

	CCH	CCH+ SCH58261	CCH+ CGS21680	CCH+ CGS21680 (KO)		
Mean ± SD	22.50 ± 4.04	11.83 ± 3.13	30.67 ± 3.27	10.67 ± 2.25		
Normality: Shapiro-Wilk normality test (W) P	(0.94) 0.64	(0.98) 0.94	(0.92) 0.51	(0.91) 0.41		
Homogeneity of Variances: Levene Statistic					F(3,20)=0.739	p=0.541
One way ANOVA					F=51.510	p=0.000

Multiple Comparisons	CCH vs. CCH+ SCH58261	CCH vs. CCH+ CGS21680	CCH vs. CCH+ CGS21680(KO)
Tukey HSD	p=0.000	p=0.002	p=0.000
LSD	p=0.000	p=0.000	p=0.000
	CCH+ SCH58261 vs. CCH+ CGS21680	CCH+ SCH58261 vs. CCH+ CGS21680 (KO)	CCH+ CGS21680 vs. CCH+ CGS21680 (KO)
Tukey HSD	p=0.000	p=0.923	p=0.000
LSD	p=0.000	p=0.539	p=0.000

Supplemental figure 4: Detection of white matter injury in the internal capsule using immumohistochemical staining for MBP after CCH. Scale bars = 50 μm.

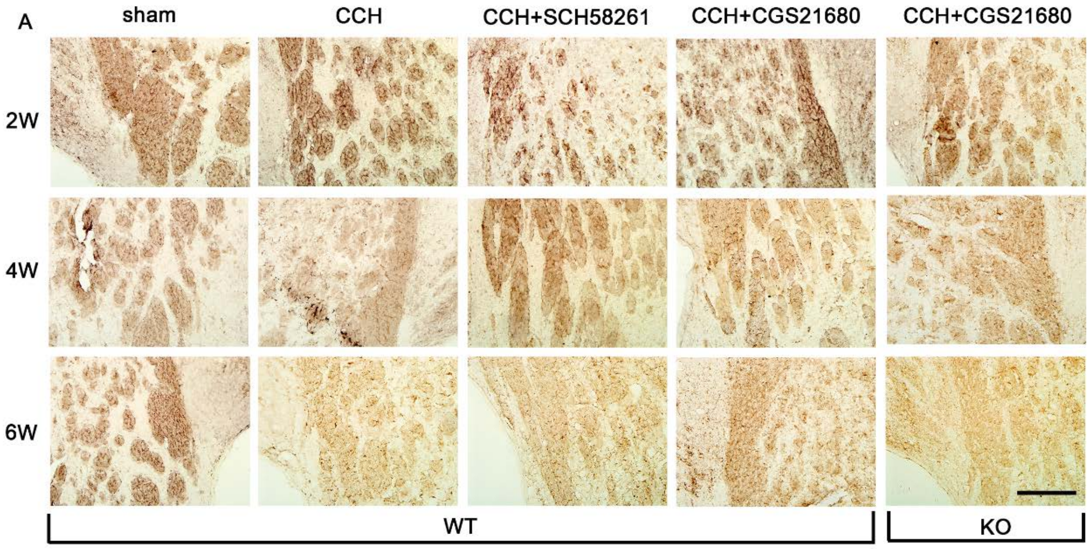

Supplemental figure 5: Original data for the statistical analysis of the MBP absorbance in the internal capsule at the end of 2nd week after CCH (for figure 1C).

2w original data	CCH	CCH+ SCH58261	CCH+ CGS21680	CCH+ CGS21680 (KO)
internal capsule	69	52	81	49
	73	45	79	47
	69	47	82	56
	68	56	84	51
	64	54	76	52
	76	62	76	46

	CCH	CCH+ SCH58261	CCH+ CGS21680	CCH+ CGS21680 (KO)		
Mean ± SD	69.84 ± 4.17	52.67 ± 6.19	79.67 ± 3.27	50.17 ± 3.66		
Normality: Shapiro-Wilk normality test (W) P	(0.96) 0.80	(0.97) 0.90	(0.92) 0.51	(0.96) 0.82		
Homogeneity of Variances: Levene Statistic					F(3,20)=0.849	p=0.483
One way ANOVA					F=59.841	p=0.000

Multiple Comparisons	CCH vs. CCH+ SCH58261	CCH vs. CCH+ CGS21680	CCH vs. CCH+ CGS21680(KO)
Tukey HSD	p=0.000	p=0.005	p=0.000
LSD	p=0.000	p=0.001	p=0.000
	CCH+ SCH58261 vs. CCH+ CGS21680	CCH+ SCH58261 vs. CCH+ CGS21680 (KO)	CCH+ CGS21680 vs. CCH+ CGS21680 (KO)
Tukey HSD	p=0.000	p=0.768	p=0.000
LSD	p=0.000	p=0.344	p=0.000

Supplemental figure 6: Original data for the statistical analysis of the MBP absorbance in the internal capsule at the end of 4th week after CCH (for figure 1C).

4w original data	CCH	CCH+ SCH58261	CCH+ CGS21680	CCH+ CGS21680 (KO)
internal capsule	54	36	68	37
	52	37	67	45
	52	41	69	44
	47	44	70	42
	48	42	68	41
	52	38	58	36

	CCH	CCH+ SCH58261	CCH+ CGS21680	CCH+ CGS21680 (KO)		
Mean ± SD	50.83 ± 2.71	39.67 ± 3.14	66.67 ± 4.37	40.83 ± 3.66		
Normality: Shapiro-Wilk normality test (W) P	(0.86) 0.18	(0.94) 0.66	(0.72) 0.33	(0.92) 0.50		
Homogeneity of Variances: Levene Statistic					F(3,20)=0.156	p=0.924
One way ANOVA					F=75.427	p=0.000

Multiple Comparisons	CCH vs. CCH+ SCH58261	CCH vs. CCH+ CGS21680	CCH vs. CCH+ CGS21680(KO)

Tukey HSD	p=0.000	p=0.000	p=0.000
LSD	p=0.000	p=0.000	p=0.000
	CCH+ SCH58261 vs. CCH+ CGS21680	CCH+ SCH58261 vs. CCH+ CGS21680 (KO)	CCH+ CGS21680 vs. CCH+ CGS21680 (KO)
Tukey HSD	p=0.000	p=0.939	p=0.000
LSD	p=0.000	p=0.573	p=0.000

Supplemental figure 7: Original data for the statistical analysis of the MBP absorbance in the internal capsule at the end of 6th week after CCH (for figure 1C).

6w original data	CCH	CCH+ SCH58261	CCH+ CGS21680	CCH+ CGS21680 (KO)
internal capsule	41	19	48	22
	31	24	58	19
	33	22	46	24
	39	23	51	24
	37	21	53	21
	41	23	46	23

	CCH	CCH+ SCH58261	CCH+ CGS21680	CCH+ CGS21680 (KO)		
Mean ± SD	37.00 ± 4.20	22.00 ± 1.79	49.50 ± 3.27	22.17 ± 1.94		
Normality: Shapiro-Wilk normality test (W) P	(0.89) 0.32	(0.93) 0.61	(0.84) 0.15	(0.91) 0.45		
Homogeneity of Variances: Levene Statistic					F(3,20)=3.125	p=0.049
One way ANOVA					F=119.357	p=0.000

Multiple Comparisons	CCH vs. CCH+ SCH58261	CCH vs. CCH+ CGS21680	CCH vs. CCH+ CGS21680(KO)
Tukey HSD	p=0.000	p=0.000	p=0.000
LSD	p=0.000	p=0.000	p=0.000
	CCH+ SCH58261 vs. CCH+ CGS21680	CCH+ SCH58261 vs. CCH+ CGS21680 (KO)	CCH+ CGS21680 vs. CCH+ CGS21680 (KO)
Tukey HSD	p=0.000	p=1.000	p=0.000
LSD	p=0.000	p=0.924	p=0.000

Supplemental figure 8: Detection of white matter injury in the optic tract using immumohistochemical staining for MBP after CCH. Scale bars = 50 μm.

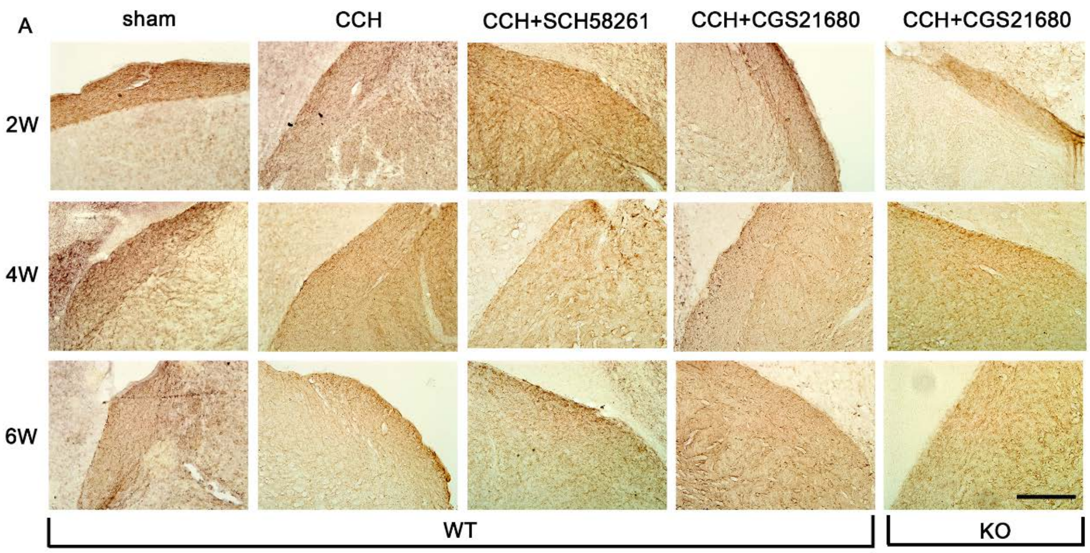

Supplemental figure 9: Original data for the statistical analysis of the MBP absorbance in the optic tract at the end of 2nd week after CCH (for figure 1D).

2w original data	CCH	CCH+ SCH58261	CCH+ CGS21680	CCH+ CGS21680 (KO)
optic tract	46	34	54	37
	39	33	62	34
	51	34	51	33
	45	36	59	32
	47	34	57	33
	42	37	55	36

	CCH	CCH+ SCH58261	CCH+ CGS21680	CCH+ CGS21680 (KO)		
Mean ± SD	45.00 ± 4.15	34.67 ± 1.51	56.33 ± 3.88	34.17 ± 1.94		
Normality: Shapiro-Wilk normality test (W) P	(0.99) 0.98	(0.87) 0.21	(0.99) 0.99	(0.91) 0.45		
Homogeneity of Variances: Levene Statistic					F(3,20)=1.767	p=0.186
One way ANOVA					F=68.597	p=0.000

Multiple Comparisons	CCH vs. CCH+ SCH58261	CCH vs. CCH+ CGS21680	CCH vs. CCH+ CGS21680(KO)
Tukey HSD	p=0.000	p=0.000	p=0.000
LSD	p=0.000	p=0.000	p=0.000
	CCH+ SCH58261 vs. CCH+ CGS21680	CCH+ SCH58261 vs. CCH+ CGS21680 (KO)	CCH+ CGS21680 vs. CCH+ CGS21680 (KO)
Tukey HSD	p=0.000	p=0.992	p=0.000
LSD	p=0.000	p=0.782	p=0.000

Supplemental figure 10: Original data for the statistical analysis of the MBP absorbance in the optic tract at the end of 4th week after CCH (for figure 1D).

4w original data	CCH	CCH+ SCH58261	CCH+ CGS21680	CCH+ CGS21680 (KO)
optic tract	36	26	45	24

	32	25	46	27
	34	24	46	26
	37	24	52	25
	33	23	42	26
	32	24	46	24

	CCH	CCH+ SCH58261	CCH+ CGS21680	CCH+ CGS21680 (KO)		
Mean ± SD	34.00 ± 2.10	24.33 ± 1.03	46.17 ± 3.25	25.33 ± 1.21		
Normality: Shapiro-Wilk normality test (W) P	(0.89) 0.32	(0.92) 0.47	(0.85) 0.16	(0.91) 0.42		
Homogeneity of Variances: Levene Statistic					F(3,20)=0.938	p=0.441
One way ANOVA					F=140.378	p=0.000

Multiple Comparisons	CCH vs. CCH+ SCH58261	CCH vs. CCH+ CGS21680	CCH vs. CCH+ CGS21680(KO)
Tukey HSD	p=0.000	p=0.000	p=0.000
LSD	p=0.000	p=0.000	p=0.000
	CCH+ SCH58261 vs. CCH+ CGS21680	CCH+ SCH58261 vs. CCH+ CGS21680 (KO)	CCH+ CGS21680 vs. CCH+ CGS21680 (KO)
Tukey HSD	p=0.000	p=0.841	p=0.000
LSD	p=0.000	p=0.417	p=0.000

Supplemental figure 11: Original data for the statistical analysis of the MBP absorbance in the optic tract at the end of 6th week after CCH (for figure 1D).

6w original data	CCH	CCH+ SCH58261	CCH+ CGS21680	CCH+ CGS21680 (KO)
optic tract	25	16	35	11
	23	14	34	15
	24	14	33	16
	21	14	32	8
	22	15	31	13
	25	11	32	15

	CCH	CCH+ SCH58261	CCH+ CGS21680	CCH+ CGS21680 (KO)		
Mean ± SD	23.33 ± 1.63	14.00 ± 1.67	32.83 ± 1.47	13.00 ± 3.03		
Normality: Shapiro-Wilk normality test (W) P	(0.92) 0.51	(0.87) 0.24	(0.96) 0.80	(0.90) 0.39		
Homogeneity of Variances: Levene Statistic					F(3,20)=1.615	p=0.218
One way ANOVA					F=122.756	p=0.000

Multiple Comparisons	CCH vs. CCH+ SCH58261	CCH vs. CCH+ CGS21680	CCH vs. CCH+ CGS21680(KO)
Tukey HSD	p=0.000	p=0.000	p=0.000
LSD	p=0.000	p=0.000	p=0.000
	CCH+ SCH58261 vs. CCH+ CGS21680	CCH+ SCH58261 vs. CCH+ CGS21680 (KO)	CCH+ CGS21680 vs. CCH+ CGS21680 (KO)
Tukey HSD	p=0.000	p=0.833	p=0.000
LSD	p=0.000	p=0.408	p=0.000

Supplemental figure 12: Immunofluorescence for astrocyte activation and YKL-40 expression at the end of 4th week post CCH. The astrocyte-specific marker GFAP was labeled with Alexa Fluor 488 (green), while the YKL-40 was labeled with Alexa Fluor 555 (red). The white pentagram, the foursquare and the solid triangle indicate the corpus callosum area, the internal capsule area, and the optic tract area, respectively. Scale bars = 20 μm.

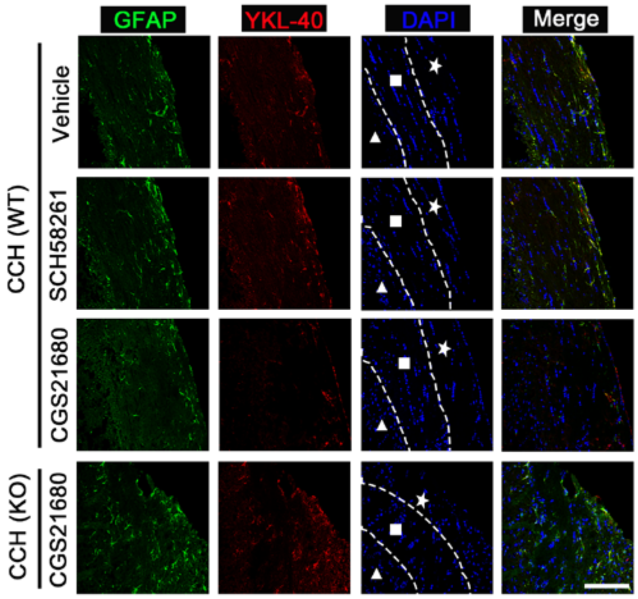

Supplemental figure 13: Immunofluorescence for astrocyte activation and YKL-40 expression at the end of 6th week post CCH. The astrocyte-specific marker GFAP was labeled with Alexa Fluor 488 (green), while the YKL-40 was labeled with Alexa Fluor 555 (red). The white pentagram, the foursquare and the solid triangle indicate the corpus callosum area, the internal capsule area, and the optic tract area, respectively. Scale bars = 20 μm.

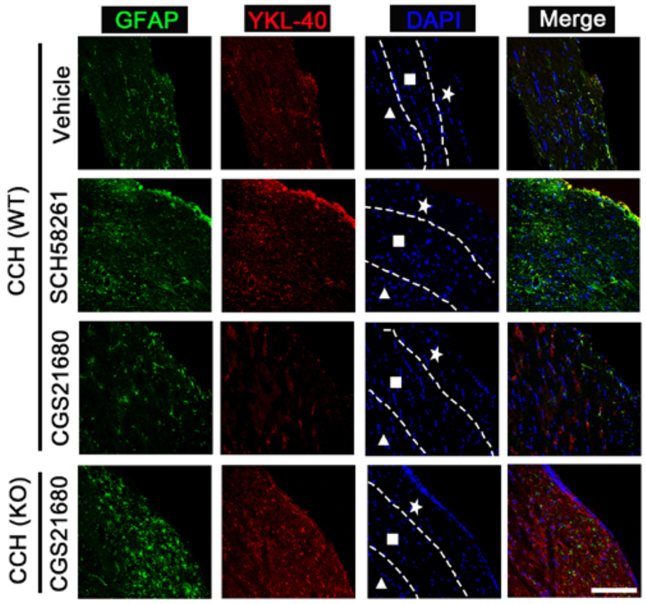

Supplemental figure 14: Original data for the statistical analysis of YKL-40^{+} cells in the corpus callosum at the end of 2nd week after CCH (for figure 2B).

2w original data	CCH	CCH+ SCH58261	CCH+ CGS21680	CCH+ CGS21680 (KO)
corpus callosum	29	79	10	71
	33	55	14	73
	26	69	17	62
	37	79	14	61
	35	66	18	74
	36	63	17	80

	CCH	CCH+ SCH58261	CCH+ CGS21680	CCH+ CGS21680 (KO)		
Mean ± SD	32.67 ± 4.32	68.50 ± 9.38	15.00 ± 2.97	70.17 ± 7.36		
Normality: Shapiro-Wilk normality test (W) P	(0.91) 0.43	(0.93) 0.56	(0.89) 0.31	(0.92) 0.49		
Homogeneity of Variances: Levene Statistic					F(3,20)=2.451	p=0.093
One way ANOVA					F=105.121	p=0.000

Multiple Comparisons	CCH vs. CCH+ SCH58261	CCH vs. CCH+ CGS21680	CCH vs. CCH+ CGS21680(KO)
Tukey HSD	p=0.000	p=0.001	p=0.000
LSD	p=0.000	p=0.000	p=0.000
	CCH+ SCH58261 vs. CCH+ CGS21680	CCH+ SCH58261 vs. CCH+ CGS21680 (KO)	CCH+ CGS21680 vs. CCH+ CGS21680 (KO)
Tukey HSD	p=0.000	p=0.970	p=0.000
LSD	p=0.000	p=0.662	p=0.000

Supplemental figure 15: Original data for the statistical analysis of YKL-40^{+} cells in the corpus callosum at the end of 4th week after CCH (for figure 2B).

4w original data	CCH	CCH+ SCH58261	CCH+ CGS21680	CCH+ CGS21680 (KO)
corpus callosum	61	104	66	103
	60	98	69	115
	76	97	55	91
	69	102	42	108
	72	94	44	99
	75	100	55	110

	CCH	CCH+ SCH58261	CCH+ CGS21680	CCH+ CGS21680 (KO)		
Mean ± SD	69.83 ± 5.71	99.17 ± 3.60	55.17 ± 11.02	104.33± 8.57		
Normality: Shapiro-Wilk normality test (W) P	(0.95) 0.74	(0.99) 0.99	(0.91) 0.46	(0.98) 0.95		
Homogeneity of Variances: Levene Statistic					F(3,20)=1.875	p=0.166
One way ANOVA					F=55.298	p=0.000

Multiple Comparisons	CCH vs. CCH+ SCH58261	CCH vs. CCH+ CGS21680	CCH vs. CCH+ CGS21680(KO)
Tukey HSD	p=0.000	p=0.018	p=0.000
LSD	p=0.000	p=0.004	p=0.000
	CCH+ SCH58261 vs. CCH+ CGS21680	CCH+ SCH58261 vs. CCH+ CGS21680 (KO)	CCH+ CGS21680 vs. CCH+ CGS21680 (KO)
Tukey HSD	p=0.000	p=0.661	p=0.000

LSD	p=0.000	p=0.262	p=0.000

Supplemental figure 16: Original data for the statistical analysis of YKL-40^{+} cells in the corpus callosum at the end of 6th week after CCH (for figure 2B).

6w original data	CCH	CCH+ SCH58261	CCH+ CGS21680	CCH+ CGS21680 (KO)
corpus callosum	105	157	67	124
	95	130	107	165
	98	149	62	129
	118	143	107	147
	125	154	83	162
	110	163	64	125

	CCH	CCH+ SCH58261	CCH+ CGS21680	CCH+ CGS21680 (KO)		
Mean ± SD	108.50± 11.57	149.33 ± 11.67	81.67 ± 20.97	142.00± 18.63		
Normality: Shapiro-Wilk normality test (W) P	(0.96) 0.82	(0.96) 0.83	(0.82) 0.09	(0.85) 0.15		
Homogeneity of Variances: Levene Statistic					F(3,20)=2.461	p=0.092
One way ANOVA					F=22.289	p=0.000

Multiple Comparisons	CCH vs. CCH+ SCH58261	CCH vs. CCH+ CGS21680	CCH vs. CCH+ CGS21680(KO)
Tukey HSD	p=0.002	p=0.044	p=0.010
LSD	p=0.000	p=0.010	p=0.002
	CCH+ SCH58261 vs. CCH+ CGS21680	CCH+ SCH58261 vs. CCH+ CGS21680 (KO)	CCH+ CGS21680 vs. CCH+ CGS21680 (KO)
Tukey HSD	p=0.000	p=0.862	p=0.000
LSD	p=0.000	p=0.444	p=0.000

Supplemental figure 17: Original data for the statistical analysis of YKL-40^{+} cells in the internal capsule at the end of 2nd week after CCH (for figure 2C).

2w original data	CCH	CCH+ SCH58261	CCH+ CGS21680	CCH+ CGS21680 (KO)
internal capsule	44	71	19	75
	43	69	25	59
	32	64	27	71
	32	81	26	81
	42	74	23	85
	42	77	16	74

	CCH	CCH+ SCH58261	CCH+ CGS21680	CCH+ CGS21680 (KO)		
Mean ± SD	39.17± 5.60	72.67 ± 6.02	22.67 ± 4.32	74.17± 9.00		
Normality: Shapiro-Wilk normality test (W) P	(0.83) 0.10	(0.99) 0.99	(0.91) 0.43	(0.95) 0.74		
Homogeneity of Variances: Levene Statistic					F(3,20)=0.552	p=0.653
One way ANOVA					F=92.953	p=0.000

Multiple Comparisons	CCH vs. CCH+ SCH58261	CCH vs. CCH+ CGS21680	CCH vs. CCH+ CGS21680(KO)
Tukey HSD	p=0.000	p=0.001	p=0.000

LSD	p=0.000	p=0.000	p=0.000
	CCH+ SCH58261 vs. CCH+ CGS21680	CCH+ SCH58261 vs. CCH+ CGS21680 (KO)	CCH+ CGS21680 vs. CCH+ CGS21680 (KO)
Tukey HSD	p=0.000	p=0.977	p=0.000
LSD	p=0.000	p=0.692	p=0.000

Supplemental figure 18: Original data for the statistical analysis of YKL-40^{+} cells in the internal capsule at the end of 4th week after CCH (for figure 2C).

4w original data	CCH	CCH+ SCH58261	CCH+ CGS21680	CCH+ CGS21680 (KO)
internal capsule	74	108	59	132
	78	112	58	127
	84	108	41	100
	65	118	56	95
	51	103	49	127
	71	96	44	116

	CCH	CCH+ SCH58261	CCH+ CGS21680	CCH+ CGS21680 (KO)		
Mean ± SD	70.50± 11.50	107.50 ± 7.53	51.17 ± 7.63	116..17 ± 15.46		
Normality: Shapiro-Wilk normality test (W) P	(0.96) 0.78	(0.98) 0.95	(0.89) 0.34	(0.88) 0.25		
Homogeneity of Variances: Levene Statistic					F(3,20)=1.816	p=0.177
One way ANOVA					F=46.497	p=0.000

Multiple Comparisons	CCH vs. CCH+ SCH58261	CCH vs. CCH+ CGS21680	CCH vs. CCH+ CGS21680(KO)
Tukey HSD	p=0.000	p=0.030	p=0.000
LSD	p=0.000	p=0.007	p=0.000
	CCH+ SCH58261 vs. CCH+ CGS21680	CCH+ SCH58261 vs. CCH+ CGS21680 (KO)	CCH+ CGS21680 vs. CCH+ CGS21680 (KO)
Tukey HSD	p=0.000	p=0.537	p=0.000
LSD	p=0.000	p=0.188	p=0.000

Supplemental figure 19: Original data for the statistical analysis of YKL-40^{+} cells in the internal capsule at the end of 6th week after CCH (for figure 2C).

6w original data	CCH	CCH+ SCH58261	CCH+ CGS21680	CCH+ CGS21680 (KO)
internal capsule	108	153	79	156
	102	177	92	161
	112	154	89	142
	91	164	63	162
	113	143	87	178
	109	159	78	186

	CCH	CCH+ SCH58261	CCH+ CGS21680	CCH+ CGS21680 (KO)		
Mean ± SD	105.83 ± 8.23	158.33 ± 11.52	81.33 ± 10.56	164.17 ± 15.75		
Normality: Shapiro-Wilk normality test (W) P	(0.86) 0.18	(0.97) 0.91	(0.90) 0.40	(0.96) 0.84		
Homogeneity of Variances: Levene Statistic					F(3,20)=0.735	p=0.543

One way ANOVA					F=69.933	p=0.000

Multiple Comparisons	CCH vs. CCH+ SCH58261	CCH vs. CCH+ CGS21680	CCH vs. CCH+ CGS21680(KO)
Tukey HSD	p=0.000	p=0.009	p=0.000
LSD	p=0.000	p=0.002	p=0.000
	CCH+ SCH58261 vs. CCH+ CGS21680	CCH+ SCH58261 vs. CCH+ CGS21680 (KO)	CCH+ CGS21680 vs. CCH+ CGS21680 (KO)
Tukey HSD	p=0.000	p=0.828	p=0.000
LSD	p=0.000	p=0.403	p=0.000

Supplemental figure 20: Original data for the statistical analysis of YKL-40^{+} cells in the optic tract at the end of 2nd week after CCH (for figure 2D).

2w original data	CCH	CCH+ SCH58261	CCH+ CGS21680	CCH+ CGS21680 (KO)
optic tract	29	40	14	38
	22	49	11	51
	22	47	10	54
	30	48	5	47
	20	56	15	45
	21	55	13	50

	CCH	CCH+ SCH58261	CCH+ CGS21680	CCH+ CGS21680 (KO)		
Mean ± SD	24.00 ± 4.34	49.173 ± 5.85	11.33 ± 3.61	47.50 ± 5.61		
Normality: Shapiro-Wilk normality test (W) P	(0.80) 0.06	(0.93) 0.60	(0.91) 0.47	(0.95) 0.75		
Homogeneity of Variances: Levene Statistic					F(3,20)=0.407	p=0.750
One way ANOVA					F=83.832	p=0.000

Multiple Comparisons	CCH vs. CCH+ SCH58261	CCH vs. CCH+ CGS21680	CCH vs. CCH+ CGS21680(KO)
Tukey HSD	p=0.000	p=0.001	p=0.000
LSD	p=0.000	p=0.000	p=0.000
	CCH+ SCH58261 vs. CCH+ CGS21680	CCH+ SCH58261 vs. CCH+ CGS21680 (KO)	CCH+ CGS21680 vs. CCH+ CGS21680 (KO)
Tukey HSD	p=0.000	p=0.936	p=0.000
LSD	p=0.000	p=0.565	p=0.000

Supplemental figure 21: Original data for the statistical analysis of YKL-40^{+} cells in the optic tract at the end of 4th week after CCH (for figure 2D).

4w original data	CCH	CCH+ SCH58261	CCH+ CGS21680	CCH+ CGS21680 (KO)
optic tract	68	107	35	119
	59	118	34	86
	67	102	45	104
	58	100	33	110
	55	85	37	111
	62	101	45	120

	CCH	CCH+ SCH58261	CCH+ CGS21680	CCH+ CGS21680 (KO)		

Mean ± SD	61.50 ± 5.17	102.17 ± 10.72	38.17 ± 5.46	108.33 ± 12.47		
Normality: Shapiro-Wilk normality test (W) P	(0.93) 0.59	(0.94) 0.68	(0.81) 0.07	(0.88) 0.27		
Homogeneity of Variances: Levene Statistic					F(3,20)=0.898	p=0.460
One way ANOVA					F=82.282	p=0.000

Multiple Comparisons	CCH vs. CCH+ SCH58261	CCH vs. CCH+ CGS21680	CCH vs. CCH+ CGS21680(KO)
Tukey HSD	p=0.000	p=0.001	p=0.000
LSD	p=0.000	p=0.000	p=0.000
	CCH+ SCH58261 vs. CCH+ CGS21680	CCH+ SCH58261 vs. CCH+ CGS21680 (KO)	CCH+ CGS21680 vs. CCH+ CGS21680 (KO)
Tukey HSD	p=0.000	p=0.645	p=0.000
LSD	p=0.000	p=0.251	p=0.000

Supplemental figure 22: Original data for the statistical analysis of YKL-40^{+} cells in the optic tract at the end of 6th week after CCH (for figure 2D).

6w original data	CCH	CCH+ SCH58261	CCH+ CGS21680	CCH+ CGS21680 (KO)
optic tract	120	137	90	136
	105	121	85	144
	93	122	73	126
	102	130	82	132
	109	145	79	112
	117	127	67	152

	CCH	CCH+ SCH58261	CCH+ CGS21680	CCH+ CGS21680 (KO)		
Mean ± SD	107.67 ± 9.95	130.33 ± 9.24	79.33 ± 8.31	133.67 ± 14.00		
Normality: Shapiro-Wilk normality test (W) P	(0.97) 0.90	(0.93) 0.56	(0.98) 0.96	(0.99) 0.99		
Homogeneity of Variances: Levene Statistic					F(3,20)=0.510	p=0.680
One way ANOVA					F=33.626	p=0.000

Multiple Comparisons	CCH vs. CCH+ SCH58261	CCH vs. CCH+ CGS21680	CCH vs. CCH+ CGS21680(KO)
Tukey HSD	p=0.007	p=0.001	p=0.002
LSD	p=0.001	p=0.000	p=0.000
	CCH+ SCH58261 vs. CCH+ CGS21680	CCH+ SCH58261 vs. CCH+ CGS21680 (KO)	CCH+ CGS21680 vs. CCH+ CGS21680 (KO)
Tukey HSD	p=0.000	p=0.947	p=0.000
LSD	p=0.000	p=0.592	p=0.000

Supplemental figure 23: Original data for the statistical analysis of *YKL-40* mRNA in the cerebrum at the end of 2nd week after CCH (for figure 2E).

2w original data	sham	CCH	CCH+ SCH58261	CCH+ CGS21680	CCH+ CGS21680 (KO)
YKL-40 mRNA	1.00	2.46	4.03	1.28	3.98
	1.00	1.75	3.70	1.48	2.81
	1.00	2.25	3.79	1.05	4.03
	1.00	1.96	4.13	0.87	3.54
	1.00	2.14	3.94	1.17	3.58

	1.00	2.73	3.24	1.21	3.49

	sham	CCH	CCH+ SCH58261	CCH+ CGS21680	CCH+ CGS21680 (KO)		
Mean ± SD	1.00 ± 0.00	2.22 ± 0.35	3.81 ± 0.32	1.18 ± 0.21	3.57 ± 0.44		
Normality: Shapiro-Wilk normality test (W) P	(1.00) 1.00	(0.99) 0.99	(0.91) 0.44	(0.99) 0.99	(0.89) 0.31		
Homogeneity of Variances: Levene Statistic						F(4,25)=2.248	p=0.093
One way ANOVA						F=111.457	p=0.000

Multiple Comparisons	Sham vs. CCH	CCH+ vs. CCH+ SCH58261	CCH vs. CCH+ CGS21680	CCH+ vs. CCH+ CGS21680 (KO)
Tukey HSD	p=0.000	p=0.000	p=0.000	p=0.000
LSD	p=0.000	p=0.000	p=0.000	p=0.000
	CCH+ SCH58261 vs. CCH+ CGS21680	CCH+ SCH58261 vs. CCH+ CGS21680 (KO)	CCH+ CGS21680 vs. CCH+ CGS21680 (KO)	
Tukey HSD	p=0.000	p=0.647	p=0.000	
LSD	p=0.000	p=0.194	p=0.000	

Supplemental figure 24: Original data for the statistical analysis of *YKL-40* mRNA in the cerebrum at the end of 4th week after CCH (for figure 2E).

4w original data	sham	CCH	CCH+ SCH58261	CCH+ CGS21680	CCH+ CGS21680 (KO)
YKL-40 mRNA	1.00	3.47	7.88	1.59	7.76
	1.00	2.98	7.55	1.71	8.05
	1.00	3.90	7.23	1.97	6.34
	1.00	3.63	8.27	1.61	7.07
	1.00	3.68	6.94	1.57	7.61
	1.00	3.33	7.58	1.84	7.24

	sham	CCH	CCH+ SCH58261	CCH+ CGS21680	CCH+ CGS21680 (KO)		
Mean ± SD	1.00 ± 0.00	3.50 ± 0.32	7.58 ± 0.47	1.72 ± 0.16	7.35 ± 0.61		
Normality: Shapiro-Wilk normality test (W) P	(1.00) 1.00	(0.97) 0.91	(0.99) 0.97	(0.88) 0.27	(0.96) 0.80		
Homogeneity of Variances: Levene Statistic						F(4,25)=4.065	p=0.011
One way ANOVA						F=400.781	p=0.000

Multiple Comparisons	Sham vs. CCH	CCH+ vs. CCH+ SCH58261	CCH vs. CCH+ CGS21680	CCH+ vs. CCH+ CGS21680 (KO)
Tukey HSD	p=0.000	p=0.000	p=0.000	p=0.000
LSD	p=0.000	p=0.000	p=0.000	p=0.000
	CCH+ SCH58261 vs. CCH+ CGS21680	CCH+ SCH58261 vs. CCH+ CGS21680 (KO)	CCH+ CGS21680 vs. CCH+ CGS21680 (KO)	
Tukey HSD	p=0.000	p=0.828	p=0.000	
LSD	p=0.000	p=0.302	p=0.000	

Supplemental figure 25: Original data for the statistical analysis of *YKL-40* mRNA in the cerebrum at the end of 6th week after CCH (for figure 2E).

6w original data	sham	CCH	CCH+ SCH58261	CCH+ CGS21680	CCH+ CGS21680 (KO)
YKL-40 mRNA	1.00	7.17	17.38	2.75	14.34
	1.00	5.52	14.28	2.24	15.36
	1.00	6.77	16.73	2.52	15.19
	1.00	5.98	14.09	3.63	12.24
	1.00	8.86	15.58	4.09	16.47
	1.00	7.24	14.50	3.07	15.01

	sham	CCH	CCH+ SCH58261	CCH+ CGS21680	CCH+ CGS21680 (KO)		
Mean ± SD	1.00 ± 0.00	6.92 ± 1.17	15.43 ± 1.38	3.05 ± 0.70	14.77 ±1.42		
Normality: Shapiro-Wilk normality test (W) P	(1.00) 1.00	(0.95) 0.72	(0.88) 0.29	(0.96) 0.79	(0.91) 0.42		
Homogeneity of Variances: Levene Statistic						F(4,25)=3.255	p=0.028
One way ANOVA						F=228.360	p=0.000

Multiple Comparisons	Sham vs. CCH	CCH+ vs. CCH+ SCH58261	CCH vs. CCH+ CGS21680	CCH+ vs. CCH+ CGS21680 (KO)
Tukey HSD	p=0.000	p=0.000	p=0.000	p=0.000
LSD	p=0.000	p=0.000	p=0.000	p=0.000
	CCH+ SCH58261 vs. CCH+ CGS21680	CCH+ SCH58261 vs. CCH+ CGS21680 (KO)	CCH+ CGS21680 vs. CCH+ CGS21680 (KO)	
Tukey HSD	p=0.000	p=0.824	p=0.000	
LSD	p=0.000	p=0.298	p=0.000	

Supplemental figure 26: The original WB blots and original data for the statistical analysis of GFAP protein in the cerebrum at the end of 6th week after CCH (for figure 3B-C).

Figure 3

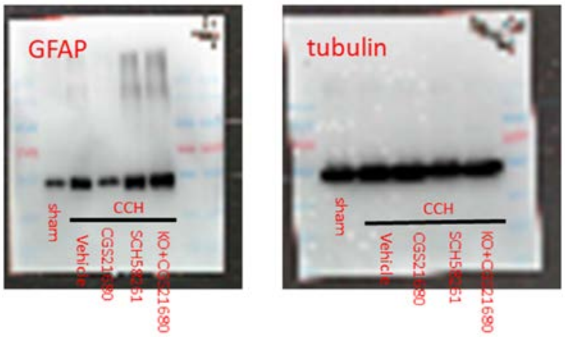

original data	sham	CCH	CCH+ CGS21680	CCH+ SCH58261	CCH+ CGS21680 (KO)
GFAP protein	1.000	4.135	1.135	6.475	7.415
(% of sham)	1.000	3.579	1.247	7.324	6.859
	1.000	4.058	0.849	5.682	6.489
	1.000	3.353	1.456	6.985	7.548
	1.000	3.876	1.357	7.148	6.358

	sham	CCH	CCH+	CCH+	CCH+		

			CGS21680	SCH58261	CGS21680 (KO)		
Mean ± SD	1.00 ± 0.00	3.80 ± 0.33	1.21 ± 0.23	6.72 ± 0.66	6.93 ±0.53		
Normality: Shapiro-Wilk normality test (W) P	(1.00) 1.00	(0.93) 0.61	(0.95) 0.75	(0.90) 0.39	(0.90) 0.41		
Homogeneity of Variances: Levene Statistic						F(4,20)=5.634	p=0.003
One way ANOVA						F=231.056	p=0.000

Multiple Comparisons	Sham vs. CCH	CCH+ vs. CCH+ CGS21680	CCH vs. CCH+ SCH58261	CCH+ vs. CCH+ CGS21680 (KO)
Tukey HSD	p=0.000	p=0.000	p=0.000	p=0.000
LSD	p=0.000	p=0.000	p=0.000	p=0.000
	CCH+ CGS21680 vs. CCH+ SCH58261	CCH+ CGS21680 vs. CCH+ CGS21680 (KO)	CCH+ SCH58261 vs. CCH+ CGS21680 (KO)	
Tukey HSD	p=0.000	p=0.000	p=0.930	
LSD	p=0.000	p=0.000	p=0.438	

Supplemental figure 27: The original WB blots and original data for the statistical analysis of STAT3 protein in the cerebrum at the end of 6th week after CCH (for figure 3B, 3D).

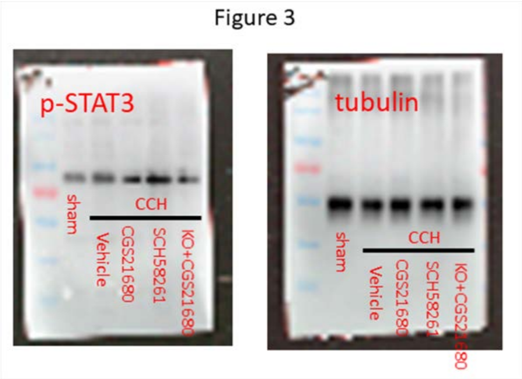

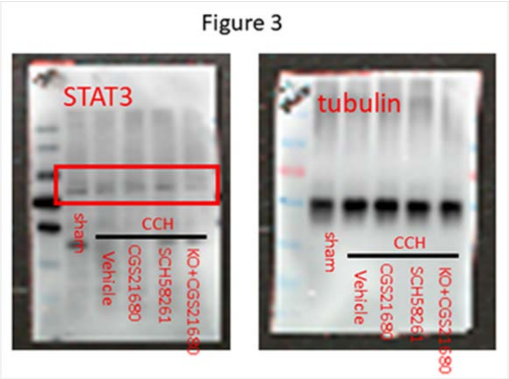

original data	sham	CCH	CCH+ CGS21680	CCH+ SCH58261	CCH+ CGS21680 (KO)
p-STAT3/STAT3 protein	1.000	2.471	0.975	4.579	3.784
(% of sham)	1.000	2.048	1.247	3.985	4.017
	1.000	3.025	1.139	4.753	4.382
	1.000	2.846	1.048	4.378	3.956
	1.000	2.846	1.475	4.175	4.285

	sham	CCH	CCH+ CGS21680	CCH+ SCH58261	CCH+ CGS21680 (KO)		
Mean ± SD	1.00 ± 0.00	2.65 ± 0.39	1.18 ± 0.20	4.37 ± 0.31	4.08 ±0.24		
Normality: Shapiro-Wilk normality test (W) P	(1.00) 1.00	(0.90) 0.38	(0.95) 0.72	(0.98) 0.94	(0.95) 0.72		
Homogeneity of Variances: Levene Statistic						F(4,20)=4.242	p=0.012
One way ANOVA						F=179.625	p=0.000

Multiple Comparisons	Sham vs. CCH	CCH+ vs. CCH+ CGS21680	CCH vs. CCH+ SCH58261	CCH+ vs. CCH+ CGS21680 (KO)
Tukey HSD	p=0.000	p=0.000	p=0.000	p=0.000

LSD	p=0.000	p=0.000	p=0.000	p=0.000
	CCH+ CGS21680 vs. CCH+ SCH58261	CCH+ CGS21680 vs. CCH+ CGS21680 (KO)	CCH+ SCH58261 vs. CCH+ CGS21680 (KO)	
Tukey HSD	p=0.000	p=0.000	p=0.434	
LSD	p=0.000	p=0.000	p=0.097	

Supplemental figure 28: The original WB blots and original data for the statistical analysis of YKL-40 protein in the cerebrum at the end of 6th week after CCH (for figure 3E-F).

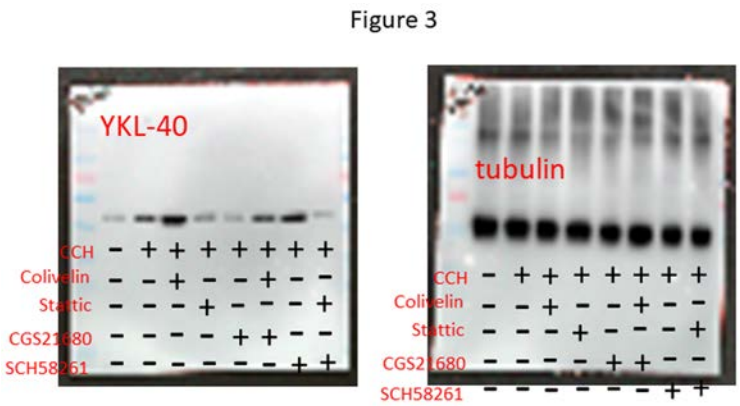

original data	sham	CCH	CCH+ Colivelin	CCH+ Stattic	CCH+ CGS21680	CCH+ CGS21680+ Colivelin	CCH+ SCH58261	CCH+ SCH58261+ Stattic
YKL-40 protein	1.000	2.167	4.278	1.247	1.048	2.348	4.675	1.378
% of sham	1.000	1.876	3.948	1.359	0.998	2.654	4.254	1.256
	1.000	2.247	4.352	1.048	0.875	2.415	4.398	1.242
	1.000	2.356	4.178	1.468	1.247	2.258	4.475	1.359
	1.000	2.451	4.487	1.358	1.365	2.789	4.568	1.225

	sham	CCH	CCH+ Colivelin	CC+ Stattic	CCH+ CGS21680	CCH+ CGS21680+Colivelin	CCH+ SCH58261	CCH+ SCH58261+ Stattic		
Mean ± SD	1.00 ± 0.00	2.22 ± 0.22	4.25 ± 0.20	1.30 ± 0.16	1.11± 0.20	2.49± 0.22	4.47± 0.16	1.29± 0.07		
Normality: Shapiro-Wilk normality test (W) P	(1.00) 1.00	(0.95) 0.70	(0.95) 0.72	(0.98) 0.93	(0.93) 0.57	(0.96) 0.79	(0.93) 0.60	(0.99) 0.99		
Homogeneity of Variances: Levene Statistic									F(7,32) =2.402	p=0.043
One way ANOVA									F=335.330	p=0.000

Multiple Comparisons	Sham vs. CCH	CCH vs. CCH+ Colivelin	CCH vs. CCH+ Stattic	CCH+ vs. CCH+ CGS21680
Tukey HSD	p=0.000	p=0.000	p=0.000	p=0.000
LSD	p=0.000	p=0.000	p=0.000	p=0.000
	CCH vs. CCH + CGS21680 +Colivelin	CCH vs. CCH+ SCH58261	CCH vs. CCH+ SCH58261 + Stattic	CCH+ Colivelin vs. CCH+ Stattic
Tukey HSD	p=0.219	p=0.000	p=0.000	p=0.000
LSD	p=0.016	p=0.000	p=0.000	p=0.000
	CCH+ CGS21680 vs. CCH+ CGS21680 +Colivelin	CCH+ SCH58261 vs. CCH+ SCH58261+ Stattic		

Tukey HSD	p=0.000	p=0.000		
LSD	p=0.000	p=0.000		

Supplemental figure 29: The original WB blots and original data for the statistical analysis of STAT3 protein in astrocytes *in vitro* (for figure 4B-C).

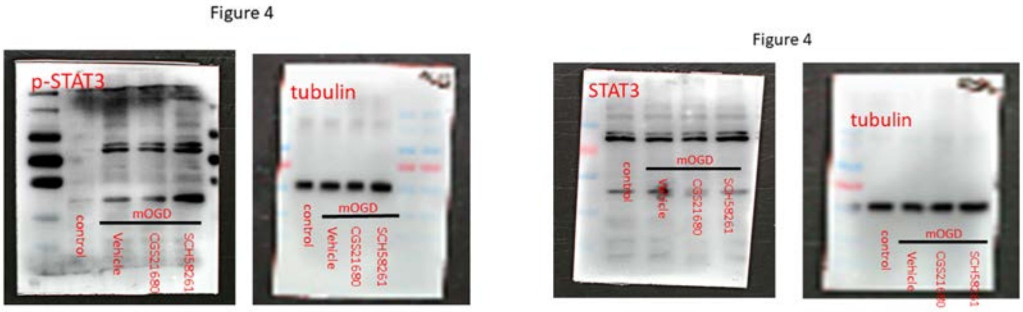

original data	control	mOGD	mOGD + CGS21680	mOGD + SCH58261
p-STAT3/STAT3 protein	1.000	3.248	2.496	4.768
(% of control)	1.000	3.648	2.578	4.598
	1.000	4.175	2.369	4.668
	1.000	3.853	2.648	4.387
	1.00	3.479	2.296	4.579

	control	mOGD	mOGD + CGS21680	mOGD + SCH58261		
Mean ± SD	1.00 ± 0.00	3.68 ± 0.35	2.48 ± 0.15	4.60 ± 0.14		
Normality: Shapiro-Wilk normality test (W) P	(1.00) 1.00	(0.99) 0.99	(0.96) 0.81	(0.96) 0.90		
Homogeneity of Variances: Levene Statistic					F(3,16)=4.918	p=0.013
One way ANOVA					F=291.607	p=0.000

Multiple Comparisons	control vs. mOGD	mOGD vs. mOGD + CGS21680	mOGD vs. mOGD + SCH58261	mOGD + CGS21680 vs. mOGD + SCH58261
Tukey HSD	p=0.000	p=0.000	p=0.000	p=0.000
LSD	p=0.000	p=0.000	p=0.000	p=0.000

Supplemental figure 30: The original WB blots and original data for the statistical analysis of YKL-40 protein in astrocytes *in vitro* (for figure 4B, 4D).

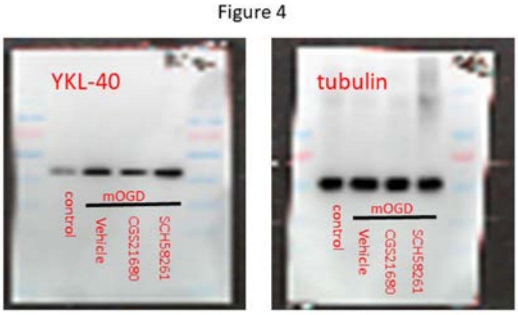

original data	control	mOGD	mOGD + CGS21680	mOGD + SCH58261

YKL-40 protein	1.000	3.697	1.154	5.847
(% of control)	1.000	4.157	1.268	6.248
	1.000	4.068	1.348	6.079
	1.000	3.986	1.256	5.879
	1.000	4.258	1.378	5.992

	control	mOGD	mOGD + CGS21680	mOGD + SCH58261		
Mean ± SD	1.00 ± 0.00	4.03 ± 0.21	1.28 ± 0.09	6.01 ± 0.16		
Normality: Shapiro-Wilk normality test (W) P	(1.00) 1.00	(0.94) 0.67	(0.95) 0.74	(0.94) 0.66		
Homogeneity of Variances: Levene Statistic					F(3,16)=3.561	p=0.038
One way ANOVA					F=1427.148	p=0.000

Multiple Comparisons	control vs. mOGD	mOGD vs. mOGD + CGS21680	mOGD vs. mOGD + SCH58261	mOGD + CGS21680 vs. mOGD + SCH58261
Tukey HSD	p=0.000	p=0.000	p=0.000	p=0.000
LSD	p=0.000	p=0.000	p=0.000	p=0.000

Supplemental figure 26: The original data for the statistical analysis of *STAT3* mRNA in astrocytes *in vitro* (for figure 4E).

original data	control	mOGD	mOGD + CGS21680	mOGD + SCH58261
STAT3 mRNA	1.000	8.478	4.687	13.597
(% of control)	1.000	9.687	3.468	12.635
	1.000	10.475	6.985	11.489
	1.000	9.542	7.635	13.549
	1.000	8.798	6.358	14.756

	control	mOGD	mOGD + CGS21680	mOGD + SCH58261		
Mean ± SD	1.00 ± 0.00	9.40 ± 0.79	5.83 ± 1.71	13.21 ± 1.22		
Normality: Shapiro-Wilk normality test (W) P	(1.00) 1.00	(0.96) 0.82	(0.94) 0.67	(0.97) 0.88		
Homogeneity of Variances: Levene Statistic					F(3,16)=6.296	p=0.005
One way ANOVA					F=107.217	p=0.000

Multiple Comparisons	control vs. mOGD	mOGD vs. mOGD + CGS21680	mOGD vs. mOGD + SCH58261	mOGD + CGS21680 vs. mOGD + SCH58261
Tukey HSD	p=0.000	p=0.001	p=0.000	p=0.000
LSD	p=0.000	p=0.000	p=0.000	p=0.000

Supplemental figure 31: The original data for the statistical analysis of *YKL-40* mRNA in astrocytes *in vitro* (for figure 4F).

original data	control	mOGD	mOGD + CGS21680	mOGD + SCH58261
YKL-40 mRNA	1.000	8.695	4.127	6.682
(% of control)	1.000	7.358	2.596	5.351
	1.000	9.145	3.557	7.496

	1.000	7.586	4.015	7.562
	1.000	8.567	3.887	5.697

	control	mOGD	mOGD + CGS21680	mOGD + SCH58261		
Mean ± SD	1.00 ± 0.00	8.27 ± 0.76	3.64 ± 0.62	6.56 ± 1.01		
Normality: Shapiro-Wilk normality test (W) P	(1.00) 1.00	(0.91) 0.45	(0.83) 0.14	(0.88) 0.33		
Homogeneity of Variances: Levene Statistic					F(3,16)=6.654	p=0.004
One way ANOVA					F=103.401	p=0.000

Multiple Comparisons	control vs. mOGD	mOGD vs. mOGD + CGS21680	mOGD vs. mOGD + SCH58261	mOGD + CGS21680 vs. mOGD + SCH58261
Tukey HSD	p=0.000	p=0.000	p=0.007	p=0.000
LSD	p=0.000	p=0.000	p=0.001	p=0.000

Supplemental figure 32: The original data for the statistical analysis of STAT3 protein in astrocytes *in vitro* (for figure 5B, 5C).

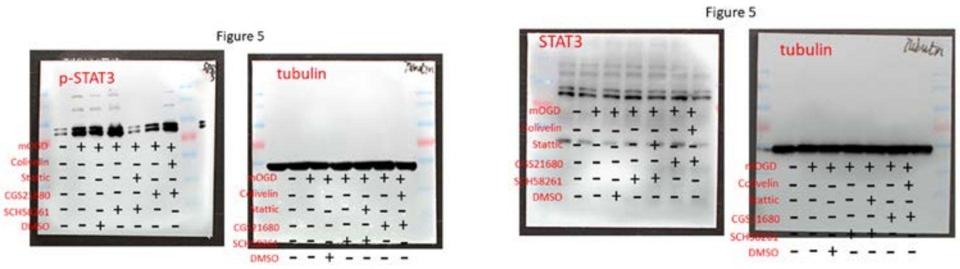

original data	control	mOGD	mOGD + DMSO	mOGD + SCH58261	CCH + SCH58261 + Stattic	mOGD + CGS21680	CCH+ CGS21680+ Colivelin
p-STAT3/STAT3 protein	1.000	4.128	3.658	6.954	1.269	1.698	4.156
(% of control)	1.000	3.589	4.257	7.149	1.578	2.247	4.021
	1.000	3.985	3.876	6.583	1.148	2.348	4.112
	1.000	3.875	3.952	6.875	1.358	1.875	3.578
	1.000	4.235	4.024	7.049	1.284	1.994	3.895

	control	mOGD	mOGD + DMSO	mOGD + SCH58261	mOGD + SCH58261 + Stattic	mOGD + CGS21680	mOGD + CGS21680 + Colivelin		
Mean ± SD	1.00 ± 0.00	3.96 ± 0.25	3.95 ± 0.22	6.92 ± 0.22	1.33 ± 0.16	2.03 ± 0.27	3.95 ± 0.23		
Normality: Shapiro-Wilk normality test (W) P	(1.00) 1.00	(0.97) 0.84	(0.99) 0.97	(0.94) 0.67	(0.93) 0.62	(0.96) 0.81	(0.89) 0.34		
Homogeneity of Variances: Levene Statistic								F(6,28)=1.741	p=0.148
One way ANOVA								F=479.731	p=0.000

Multiple Comparisons	control vs. mOGD	control vs. mOGD + DMSO	mOGD vs. mOGD + DMSO	mOGD vs. mOGD + SCH58261
Tukey HSD	p=0.000	p=0.000	p=1.000	p=0.000
LSD	p=0.000	p=0.000	p=0.946	p=0.000
	mOGD vs. mOGD + SCH58261 + Stattic	mOGD vs. mOGD + CGS21680	mOGD vs. mOGD + CGS21680+ Colivelin	mOGD + DMSO vs. mOGD + SCH58261

Tukey HSD	p=0.000	p=0.000	p=1.000	p=0.000
LSD	p=0.000	p=0.000	p=0.940	p=0.000
	mOGD + DMSO vs. mOGD + SCH58261 + Stattic	mOGD + DMSO vs. mOGD + CGS21680	mOGD + DMSO vs. mOGD + CGS21680+ Colivelin	mOGD + SCH58261 vs. mOGD + SCH58261 + Stattic
Tukey HSD	p=0.000	p=0.000	p=1.000	p=0.000
LSD	p=0.000	p=0.000	p=0.994	p=0.000
	mOGD + CGS21680 vs. mOGD + CGS21680+ Colivelin			
Tukey HSD	p=0.000			
LSD	p=0.000			

Supplemental figure 33: The original data for the statistical analysis of YKL-40 protein in astrocytes *in vitro* (for figure 5B, 5D).

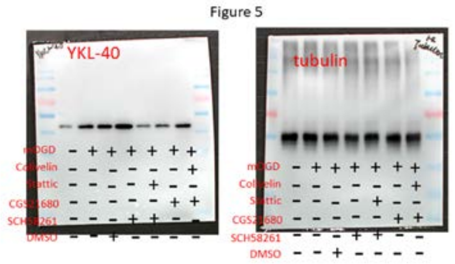

original data	control	mOGD	mOGD + DMSO	mOGD + SCH58261	CCH + SCH58261 + Stattic	mOGD + CGS21680	CCH+ CGS21680+ Colivelin
YKL-40 protein	1.000	3.156	2.986	5.479	1.146	1.795	2.876
(% of control)	1.000	2.978	3.263	6.325	0.978	2.016	3.246
	1.000	3.258	3.028	5.896	1.245	2.148	3.148
	1.000	3.025	3.147	5.741	1.345	1.986	3.058
	1.000	2.879	2.857	5.995	1.187	1.853	3.369

	control	mOGD	mOGD + DMSO	mOGD + SCH58261	mOGD + SCH58261 + Stattic	mOGD + CGS21680	mOGD + CGS21680 + Colivelin		
Mean ± SD	1.00 ± 0.00	3.06 ± 0.15	3.06 ± 0.16	5.89 ± 0.31	1.18 ± 0.14	1.96 ± 0.14	3.14 ± 0.19		
Normality: Shapiro-Wilk normality test (W) P	(1.00) 1.00	(0.98) 0.91	(0.99) 0.97	(0.99) 0.99	(0.98) 0.92	(0.94) 0.84	(0.99) 0.99		
Homogeneity of Variances: Levene Statistic								F(6,28)=2.113	p=0.083
One way ANOVA								F=438.915	p=0.000

Multiple Comparisons	control vs. mOGD	control vs. mOGD + DMSO	mOGD vs. mOGD + DMSO	mOGD vs. mOGD + SCH58261
Tukey HSD	p=0.000	p=0.000	p=1.000	p=0.000
LSD	p=0.000	p=0.000	p=0.979	p=0.000
	mOGD vs. mOGD + SCH58261 + Stattic	mOGD vs. mOGD + CGS21680	mOGD vs. mOGD + CGS21680+ Colivelin	mOGD + DMSO vs. mOGD + SCH58261
Tukey HSD	p=0.000	p=0.000	p=0.990	p=0.000
LSD	p=0.000	p=0.000	p=0.477	p=0.000
	mOGD + DMSO vs. mOGD + SCH58261 +	mOGD + DMSO vs. mOGD + CGS21680	mOGD + DMSO vs. mOGD + CGS21680+	mOGD + SCH58261 vs. mOGD + SCH58261 +

	Stattic		Colivelin	Stattic
Tukey HSD	p=0.000	p=0.000	p=0.988	p=0.000
LSD	p=0.000	p=0.000	p=0.461	p=0.000
	mOGD + CGS21680 vs. mOGD + CGS21680+ Colivelin			
Tukey HSD	p=0.000			
LSD	p=0.000			

Supplemental figure 34: The original data for the statistical analysis of *STAT3* mRNA in astrocytes *in vitro* (for figure 5E).

original data	control	mOGD	mOGD + DMSO	mOGD + SCH58261	CCH + SCH58261 + Stattic	mOGD + CGS21680	CCH+ CGS21680+ Colivelin
STAT3 mRNA	1.00	7.162	7.146	10.687	3.758	4.479	6.495
(% of control)	1.00	5.495	6.593	9.356	2.697	4.587	9.556
	1.00	9.774	9.685	11.458	2.489	5.147	8.675
	1.00	8.698	7.224	12.447	3.227	3.564	7.482
	1.00	8.247	8.762	9.578	3.048	5.228	8.357

	control	mOGD	mOGD + DMSO	mOGD + SCH58261	mOGD + SCH58261 + Stattic	mOGD + CGS21680	mOGD + CGS21680 + Colivelin		
Mean ± SD	1.00 ± 0.00	7.88 ± 1.63	7.88 ± 1.29	10.71 ± 1.29	3.04 ± 0.49	4.60 ± 0.67	8.11 ± 1.17		
Normality: Shapiro-Wilk normality test (W) P	(1.00) 1.00	(0.98) 0.91	(0.90) 0.40	(0.94) 0.67	(0.97) 0.88	(0.90) 0.42	(0.99) 0.97		
Homogeneity of Variances: Levene Statistic								F(6,28)=3.542	p=0.010
One way ANOVA								F=50.144	p=0.000

Multiple Comparisons	control vs. mOGD	control vs. mOGD + DMSO	mOGD vs. mOGD + DMSO	mOGD vs. mOGD + SCH58261
Tukey HSD	p=0.000	p=0.000	p=1.000	p=0.004
LSD	p=0.000	p=0.000	p=0.992	p=0.000
	mOGD vs. mOGD + SCH58261 + Stattic	mOGD vs. mOGD + CGS21680	mOGD vs. mOGD + CGS21680+ Colivelin	mOGD + DMSO vs. mOGD + SCH58261
Tukey HSD	p=0.000	p=0.000	p=1.000	p=0.004
LSD	p=0.000	p=0.000	p=0.728	p=0.000
	mOGD + DMSO vs. mOGD + SCH58261 + Stattic	mOGD + DMSO vs. mOGD + CGS21680	mOGD + DMSO vs. mOGD + CGS21680+ Colivelin	mOGD + SCH58261 vs. mOGD + SCH58261 + Stattic
Tukey HSD	p=0.000	p=0.001	p=1.000	p=0.000
LSD	p=0.000	p=0.000	p=0.461	p=0.000
	mOGD + CGS21680 vs. mOGD + CGS21680+ Colivelin			
Tukey HSD	p=0.000			
LSD	p=0.000			

Supplemental figure 35: The original data for the statistical analysis of *YKL-40* mRNA in astrocytes *in vitro* (for figure 5F).

original data	control	mOGD	mOGD + DMSO	mOGD + SCH58261	CCH + SCH58261	mOGD + CGS21680	CCH+ CGS21680+

					+ Stattic		Colivelin
YKL-40 mRNA	1.00	10.268	8.675	14.935	3.596	6.482	9.563
(% of control)	1.00	9.367	11.468	12.745	5.247	4.915	7.675
	1.00	12.475	10.576	15.375	4.578	5.742	10.475
	1.00	11.885	9.346	10.592	5.358	6.143	8.546
	1.00	8.942	9.462	14.579	5.247	5.813	9.887

	control	mOGD	mOGD + DMSO	mOGD + SCH58261	mOGD + SCH58261 + Stattic	mOGD + CGS21680	mOGD + CGS21680 + Colivelin		
Mean ± SD	1.00 ± 0.00	10.59 ± 1.54	9.91 ± 1.11	13.65 ± 1.98	4.81 ± 0.74	5.82 ± 0.58	9.23 ± 1.12		
Normality: Shapiro-Wilk normality test (W) P	(1.00) 1.00	(0.92) 0.50	(0.94) 0.69	(0.88) 0.30	(0.80) 0.09	(0.95) 0.72	(0.96) 0.80		
Homogeneity of Variances: Levene Statistic								F(6,28)=5.222	p=0.001
One way ANOVA								F=64.868	p=0.000

Multiple Comparisons	control vs. mOGD	control vs. mOGD + DMSO	mOGD vs. mOGD + DMSO	mOGD vs. mOGD + SCH58261
Tukey HSD	p=0.000	p=0.000	p=0.967	p=0.005
LSD	p=0.000	p=0.000	p=0.367	p=0.000
	mOGD vs. mOGD + SCH58261 + Stattic	mOGD vs. mOGD + CGS21680	mOGD vs. mOGD + CGS21680+ Colivelin	mOGD + DMSO vs. mOGD + SCH58261
Tukey HSD	p=0.000	p=0.000	p=0.542	p=0.000
LSD	p=0.000	p=0.000	p=0.078	p=0.000
	mOGD + DMSO vs. mOGD + SCH58261 + Stattic	mOGD + DMSO vs. mOGD + CGS21680	mOGD + DMSO vs. mOGD + CGS21680+ Colivelin	mOGD + SCH58261 vs. mOGD + SCH58261 + Stattic
Tukey HSD	p=0.000	p=0.000	p=0.968	p=0.000
LSD	p=0.000	p=0.000	p=0.371	p=0.000
	mOGD + CGS21680 vs. mOGD + CGS21680+ Colivelin			
Tukey HSD	p=0.001			
LSD	p=0.000			

Supplemental figure 36: The original data for the statistical analysis of inflammatory cytokines IL-1β in the supernatant of primary astrocytes cultured with different treatments *in vitro* (for figure 6A).

original data	control	mOGD	mOGD + DMSO	mOGD + SCH58261	CCH + SCH58261 + Stattic	mOGD + CGS21680	CCH+ CGS21680+ Colivelin
IL-1β	3.697	46.857	44.687	64.395	21.957	14.683	54.716
(% of control)	4.258	35.697	45.234	57.149	18.647	24.715	42.587
	2.371	42.597	41.597	60.789	25.749	22.748	58.346
	2.987	51.975	48.215	68.375	22.385	18.357	42.687
	3.024	58.261	42.475	52.317	17.685	15.674	39.586

	control	mOGD	mOGD + DMSO	mOGD + SCH58261	mOGD + SCH58261 + Stattic	mOGD + CGS21680	mOGD + CGS21680 + Colivelin		
Mean ± SD	3.27 ±	47.08 ±	44.44 ±	60.61 ±	21.28 ±	19.24 ±	47.58 ±		

	0.73	8.64	2.59	6.23	3.22	4.37	8.36		
Normality: Shapiro-Wilk normality test (W) P	(0.97) 0.84	(0.99) 0.99	(0.95) 0.77	(0.99) 0.99	(0.95) 0.70	(0.92) 0.51	(0.85) 0.19		
Homogeneity of Variances: Levene Statistic								F(6,28)=4.229	p=0.004
One way ANOVA								F=66.026	p=0.000

Multiple Comparisons	control vs. mOGD	control vs. mOGD + DMSO	mOGD vs. mOGD + DMSO	mOGD vs. mOGD + SCH58261
Tukey HSD	p=0.000	p=0.000	p=0.988	p=0.011
LSD	p=0.000	p=0.000	p=0.464	p=0.001
	mOGD vs. mOGD + SCH58261 + Stattic	mOGD vs. mOGD + CGS21680	mOGD vs. mOGD + CGS21680+ Colivelin	mOGD + DMSO vs. mOGD + SCH58261
Tukey HSD	p=0.000	p=0.000	p=1.000	p=0.002
LSD	p=0.000	p=0.000	p=0.887	p=0.000
	mOGD + DMSO vs. mOGD + SCH58261 + Stattic	mOGD + DMSO vs. mOGD + CGS21680	mOGD + DMSO vs. mOGD + CGS21680+ Colivelin	mOGD + SCH58261 vs. mOGD + SCH58261 + Stattic
Tukey HSD	p=0.000	p=0.000	p=0.972	p=0.000
LSD	p=0.000	p=0.000	p=0.383	p=0.000
	mOGD + CGS21680 vs. mOGD + CGS21680+ Colivelin			
Tukey HSD	p=0.000			
LSD	p=0.000			

Supplemental figure 37: The original data for the statistical analysis of inflammatory cytokines TNF-α in the supernatant of primary astrocytes cultured with different treatments *in vitro* (for figure 6B).

original data	control	mOGD	mOGD + DMSO	mOGD + SCH58261	CCH + SCH58261 + Stattic	mOGD + CGS21680	CCH+ CGS21680+ Colivelin
TNF-α	5.697	72.587	55.214	87.246	34.879	36.251	54.156
(% of control)	4.156	60.154	52.156	74.663	45.627	30.459	65.987
	3.558	58.746	69.563	82.965	39.518	45.362	60.536
	2.597	70.524	57.452	79.453	27.463	31.598	71.486
	5.694	69.356	62.593	85.621	31.665	28.457	75.952

	control	mOGD	mOGD + DMSO	mOGD + SCH58261	mOGD + SCH58261 + Stattic	mOGD + CGS21680	mOGD + CGS21680 + Colivelin		
Mean ± SD	4.34 ± 1.36	66.27 ± 6.35	59.40 ± 6.84	81.99 ± 5.05	35.83 ± 7.03	34.43 ± 6.75	65.62 ± 8.64		
Normality: Shapiro-Wilk normality test (W) P	(0.90) 0.42	(0.85) 0.19	(0.95) 0.75	(0.95) 0.76	(0.99) 0.97	(0.88) 0.29	(0.98) 0.95		
Homogeneity of Variances: Levene Statistic								F(6,28)=1.664	p=0.167
One way ANOVA								F=85.144	p=0.000

Multiple Comparisons	control vs. mOGD	control vs. mOGD + DMSO	mOGD vs. mOGD + DMSO	mOGD vs. mOGD + SCH58261
Tukey HSD	p=0.000	p=0.000	p=0.617	p=0.009
LSD	p=0.000	p=0.000	p=0.099	p=0.001
	mOGD vs. mOGD +	mOGD vs. mOGD +	mOGD vs. mOGD +	mOGD + DMSO vs.

	SCH58261 + Stattic	CGS21680	CGS21680+ Colivelin	mOGD + SCH58261
Tukey HSD	p=0.000	p=0.000	p=1.000	p=0.000
LSD	p=0.000	p=0.000	p=0.873	p=0.000
	mOGD + DMSO vs. mOGD + SCH58261 + Stattic	mOGD + DMSO vs. mOGD + CGS21680	mOGD + DMSO vs. mOGD + CGS21680+ Colivelin	mOGD + SCH58261 vs. mOGD + SCH58261 + Stattic
Tukey HSD	p=0.000	p=0.000	p=0.716	p=0.000
LSD	p=0.000	p=0.000	p=0.133	p=0.000
	mOGD + CGS21680 vs. mOGD + CGS21680+ Colivelin			
Tukey HSD	p=0.000			
LSD	p=0.000			

Supplemental figure 38: The original data for the statistical analysis of inflammatory cytokines IL-6 in the supernatant of primary astrocytes cultured with different treatments *in vitro* (for figure 6C).

original data	control	mOGD	mOGD + DMSO	mOGD + SCH58261	CCH + SCH58261 + Stattic	mOGD + CGS21680	CCH+ CGS21680+ Colivelin
IL-6	0.648	32.574	27.541	47.695	19.365	14.362	36.596
(% of control)	2.358	29.695	35.624	53.562	22.157	15.986	30.475
	1.467	38.167	30.996	38.561	14.539	21.369	32.165
	2.018	33.584	24.756	40.963	23.687	19.475	41.578
	3.629	30.269	29.658	41.869	25.264	20.458	29.547

	control	mOGD	mOGD + DMSO	mOGD + SCH58261	mOGD + SCH58261 + Stattic	mOGD + CGS21680	mOGD + CGS21680 + Colivelin		
Mean ± SD	2.02 ± 1.11	32.86 ± 3.37	29.72 ± 4.06	44.53 ± 6.06	21.00 ± 4.22	18.33 ± 3.01	34.07 ± 4.99		
Normality: Shapiro-Wilk normality test (W) P	(0.99) 0.97	(0.91) 0.46	(0.99) 0.96	(0.91) 0.50	(0.94) 0.65	(0.91) 0.45	(0.90) 0.41		
Homogeneity of Variances: Levene Statistic								F(6,28)=2.089	p=0.087
One way ANOVA								F=55.935	p=0.000

Multiple Comparisons	control vs. mOGD	control vs. mOGD + DMSO	mOGD vs. mOGD + DMSO	mOGD vs. mOGD + SCH58261
Tukey HSD	p=0.000	p=0.000	p=0.884	p=0.002
LSD	p=0.000	p=0.000	p=0.236	p=0.000
	mOGD vs. mOGD + SCH58261 + Stattic	mOGD vs. mOGD + CGS21680	mOGD vs. mOGD + CGS21680+ Colivelin	mOGD + DMSO vs. mOGD + SCH58261
Tukey HSD	p=0.002	p=0.000	p=0.999	p=0.000
LSD	p=0.000	p=0.000	p=0.643	p=0.000
	mOGD + DMSO vs. mOGD + SCH58261 + Stattic	mOGD + DMSO vs. mOGD + CGS21680	mOGD + DMSO vs. mOGD + CGS21680+ Colivelin	mOGD + SCH58261 vs. mOGD + SCH58261 + Stattic
Tukey HSD	p=0.032	p=0.002	p=0.634	p=0.000
LSD	p=0.002	p=0.000	p=0.104	p=0.000
	mOGD + CGS21680 vs. mOGD + CGS21680+ Colivelin			

Tukey HSD	p=0.000			
LSD	p=0.000			

Supplemental figure 39: *In vitro* cytotoxicity evaluation of CGS21680 and SCH58261 in astrocyte. Cell Counting Kit-8 (CCK-8) was used to assess cytotoxicity of CGS21680 (A) and SCH58261 (B) in primary astrocyte. Cells were cultured in 96-well plates (1 × 10^4 cells per well) in 100 μL of DMEM containing 10% FBS for 24 h. Then cells were treated with various doses of CGS21680 and SCH58261 (from 0, 2, 5, 10, 20, 50, 100, 200, 500, to 1000 nM /mL) for another 24 h. Cell viability was quantified by CCK-8 assay. Data are mean ± SD (n = 3).

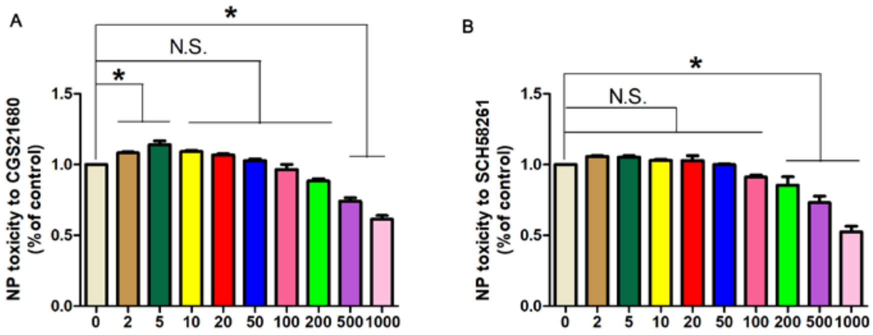

Supplemental figure 40: The original data for the statistical analysis of cell cytotoxicity for CGS21680 in primary astrocytes (for Supplemental figure 39 A).

original data	0	2	5	10	20	50	100	200	500	1000
(% of control)	1.000	1.112	1.142	1.075	1.051	1.037	0.931	0.911	0.777	0.646
	1.000	1.105	1.092	1.098	1.067	1.003	1.035	0.855	0.751	0.567
	1.000	1.099	1.187	1.104	1.083	1.045	0.931	0.888	0.693	0.632

	0	2	5	10	20	50	100	200	500	1000		
Mean ± SD	1.00 ± 1.00	1.11 ± 0.01	1.14 ± 0.05	1.09 ± 0.15	1.07 ± 0.16	1.03 ± 0.02	0.97 ± 0.06	0.88 ± 0.03	0.74 ± 0.04	0.61 ± 0.04		
Normality: Shapiro-Wilk normality test (W) P	(1.00) 1.00	(0.99) 0.92	(0.99) 0.94	(0.90) 0.38	(1.00) 1.00	(0.89) 0.34	(0.90) 0.39	(0.99) 0.80	(0.95) 0.59	(0.88) 0.32		
Homogeneity of Variances: Levene Statistic											F(9,20) =3.027	p=0.019
One way ANOVA											F=77.2 65	p=0.000

Multiple Comparisons	0 vs.2	0 vs. 5	0 vs. 10	0 vs. 20	0 vs. 50
Tukey HSD	p=0.027	p=0.002	p=0.071	p=0.351	p=0.987
LSD	p=0.001	p=0.000	p=0.003	p=0.024	p=0.317
	0 vs. 100	0 vs. 200	0 vs. 500	0 vs. 1000	
Tukey HSD	p=0.954	p=0.012	p=0.000	p=0.000	
LSD	p=0.226	p=0.000	p=0.000	p=0.000	

Supplemental figure 41: The original data for the statistical analysis of cell cytotoxicity for SCH58261 in primary astrocytes (for Supplemental figure 39 B).

original	0	2	5	10	20	50	100	200	500	1000

data										
(% of control)	1.000	1.065	1.070	1.016	1.094	0.998	0.880	0.932	0.759	0.448
	1.000	1.060	1.041	1.045	0.980	1.011	0.926	0.740	0.793	0.579
	1.000	1.050	1.047	1.027	1.012	0.989	0.927	0.891	0.639	0.547

	0	2	5	10	20	50	100	200	500	1000		
Mean ± SD	1.00 ± 0.00	1.06 ± 0.01	1.05 ± 0.02	1.03 ± 0.01	1.03 ± 0.06	1.00 ± 0.01	0.91 ± 0.03	0.85 ± 0.10	0.73 ± 0.08	0.52 ± 0.07		
Normality: Shapiro-Wilk normality test (W) P	(1.00) 1.00	(0.96) 0.64	(0.90) 0.38	(0.98) 0.74	(0.94) 0.53	(0.99) 0.80	(0.90) 0.40	(0.90) 0.39	(0.91) 0.40	(0.92) 0.45		
Homogeneity of Variances: Levene Statistic											F(9,20) =5.328	p=0.051
One way ANOVA											F=34.331	p=0.000

Multiple Comparisons	0 vs.2	0 vs. 5	0 vs. 10	0 vs. 20	0 vs. 50
Tukey HSD	p=0.917	p=0.952	p=0.999	p=0.999	p=1.000
LSD	p=0.180	p=0.223	p=0.489	p=0.502	p=0.992
	0 vs. 100	0 vs. 200	0 vs. 500	0 vs. 1000	
Tukey HSD	p=0.532	p=0.057	p=0.000	p=0.000	
LSD	p=0.046	p=0.002	p=0.000	p=0.000	
